# PoRal2 Is Involved in Appressorium Formation and Virulence *via* Pmk1 MAPK Pathways in the Rice Blast Fungus *Pyricularia oryzae*

**DOI:** 10.3389/fpls.2021.702368

**Published:** 2021-09-13

**Authors:** Yingmin Qu, Jing Wang, Pengyun Huang, Xiaohong Liu, Jianping Lu, Fu-Cheng Lin

**Affiliations:** ^1^State Key Laboratory for Managing Biotic and Chemical Threats to the Quality and Safety of Agro-Products, Institute of Biotechnology, Zhejiang University, Hangzhou, China; ^2^College of Life Sciences, Zhejiang University, Hangzhou, China; ^3^State Key Laboratory for Managing Biotic and Chemical Threats to the Quality and Safety of Agro-Products, Institute of Plant Protection and Microbiology, Zhejiang Academy of Agricultural Sciences, Hangzhou, China

**Keywords:** *Pyricularia oryzae*, *Magnaporthe oryzae*, sporulation, Pmk1 MAPK pathway, virulence, cAMP-PKA pathway, appressorium formation

## Abstract

*Pyricularia oryzae* is an important plant pathogenic fungus that can severely damage rice and wheat crops, leading to significant reductions in crop productivity. To penetrate into and invade tissues of its plant host, this fungus relies on an invasive structure known as an appressorium. Appressorium formation is rigorously regulated by the cAMP-PKA and Pmk1 MAPK pathways. Here, we identified PoRal2, a homologous protein of *Schizosaccharomyces pombe* Ral2, and characterized its roles in fungal development and virulence in *P. oryzae*. PoRal2 contains N-terminal kelch repeats and C-terminal BTB domains. PoRal2 is involved in sporulation, aerial hypha and conidiophore differentiation, appressorium formation, plant penetration, and virulence. During appressorium formation, ∆*Poral2* mutants generate appressoria with long germ tubes on hydrophobic surfaces. ∆*Poral2* mutants exhibited a defective response to exogenous cAMP and the activated *RAS2*^G18V^ on a hydrophilic surface, indicating impairment in the cAMP-PKA or Pmk1 MAPK signaling pathways. Deletion of *PoRAL2* leads to lowered Pmk1 phosphorylation level in the mutant. Moreover, PoRal2 is found to interact with Scd1, Smo1, and Mst50, which are involved in activation of Pmk1. In addition, the expression levels of *MPG1*, *WISH*, and *PDEH* in the cAMP-PKA pathway, *RAS2* in both the cAMP-PKA and Pmk1 MAPK pathways, and melanin biosynthesis genes (*ALB1*, *BUF1*, and *RSY1*) were significantly down-regulated in the ∆*Poral2*. Therefore, PoRal2 is involved in fungal development and virulence by its crosstalk in the cAMP-PKA and Pmk1 MAPK signaling pathways.

## Introduction

*Pyricularia oryzae* (synonym *Magnaporthe oryzae*) is a plant pathogenic fungus that causes rice blast disease, leading to destructive production losses in rice and wheat crops worldwide. After a conidium (asexual spore) lands on a leaf, under favorable conditions, the conidium will tightly bind to the hydrophobic leaf surface, after which a germ tube develops from the pyriform conidium base. Subsequently, the germ tube hooks, swells at the tip, and finally differentiates into a dome-shaped appressorium (Talbot, 2003; Ryder and Talbot, 2015). During appressorium maturation, glycogen and lipids gradually translocate from the conidium to the appressorium, and glycerol accumulates in the appressorial cell which generates turgor pressure as high as 8.0MPa (de Jong et al., 1997; Thines et al., 2000). The immense turgor pressure allows a structure referred to as the penetration peg, produced by the appressorium, to penetrate into the leaf cuticle. Subsequently, an invasive hypha differentiates into bulbous and branched secondary hyphae which spread intercellularly and intracellularly through host tissues (Kankanala et al., 2007). Finally, necrotic blast lesions, producing abundant conidiophores and conidia, emerge on the infected rice surface.

Ral2, identified from the fungus *Schizosaccharomyces pombe*, is a Ras-like protein in the Ras1-Scd pathway that has three kelch repeats at N-terminal end and has roles in controlling cell morphology, cell conjugation, sporulation, and interactions with Gef1 and the SCF ubiquitin ligase complex subunit Skp1 proteins (Fukui et al., 1989; Tafforeau et al., 2006; Vo et al., 2016). In *S. pombe*, Ral2 is supposed to function closely (in proximity) with Ras1 and is involved in Ras1 activation. It was found that expression of the activated form of the *RAS1* allele, *RAS1*^Val-17^, restored the rod-like cell morphology and mating factor response defects found in the *ral2* mutant (Fukui et al., 1989). RAS proteins are small GTP-binding proteins which respond to external stimuli and activate various downstream signaling pathways by the transition of RAS protein conformations between the inactive GDP-bound and active GTP-bound forms (Milburn et al., 1990). The two RAS proteins in *Saccharomyces cerevisiae*, Ras1 and Ras2, regulate the cAMP-PKA signaling pathway by affecting the activity of adenylate cyclase (Toda et al., 1985; Belotti et al., 2012). In *P. oryzae*, Ras1 merely regulates conidiation while Ras2 is essential for its survival. Moreover, Ras2 was found to function upstream of the cAMP-PKA and Pmk1 MAPK signaling pathways (Zhou et al., 2014). During appressorium formation, G protein-coupled receptors (GPCRs) recognize hydrophobic surface signals and then activate the G protein signaling pathway, which controls the crosstalk of the cAMP-PKA and Pmk1 MAPK signaling pathways in *P. oryzae* (Ebbole, 2007; Li et al., 2012a; McDonough and Rodriguez, 2012; Kou and Naqvi, 2016). Deleting the upstream components of cAMP pathway, such as *MPG1*, *PTH11*, *MAGB*, and *CPKA*, led to appressorium formation defects that could be recovered by exogenous application of cAMP (Talbot et al., 1996; Liu and Dean, 1997; Xu et al., 1997; DeZwaan et al., 1999). However, mutants with defects in the Pmk1 MAPK pathway like ∆*mst11*, ∆*mst7*, and ∆*pmk1* cannot form appressoria and could not be recovered by exogenous cAMP (Xu and Hamer, 1996; Zhao et al., 2005). These results confirmed that both the cAMP-PKA and Pmk1 MAPK signaling pathways are essential for appressorium formation.

In this study, we characterized in *P. oryzae*, the biological role of PoRal2, a kelch and BTB domain protein, for which functions have not yet been revealed in filamentous fungi. PoRal2 is involved in aerial hyphal differentiation, spore production, appressorium formation, and fungal virulence. Deletion of *PoRAL2* led to increased germ tube extension during appressorium formation, attenuated sensitivity to exogenous cAMP and the activated Ras2^G18V^, and reduced Pmk1 phosphorylation. PoRal2 plays roles in cAMP-PKA and Pmk1 MAPK signaling pathways by interacting with Mst50, Scd1, Smo1, and Gef1 in *P. oryzae*.

## Materials and Methods

### Gene Deletion, Complementation, and Expression of Fluorescent Fusion Proteins

The 1.0–1.2kb upstream and downstream DNA fragments of *PoRAL2* (GenBank: XP_003715831.1), amplified from the wild-type *P. oryzae* 70–15 strain genome, using two primer sets (Up-F/Up-R and Dn-F/Dn-R) and a hygromycin B phosphotransferase gene (*HPH*) fragment (cloned using primers *HPH*-F and *HPH*-R) were fused into a knockout cassette in a *Hin*dIII and *Sal*I-linearized pKO3A vector (Yan et al., 2019) with a fusion enzyme (Vazyme Biotech, China). The knockout cassette was transformed into *P. oryzae* conidia of the wild-type strain 70–15 *via Agrobacterium tumefaciens*-mediated transformation (ATMT) method as previously described (Lu et al., 2014; Yan et al., 2019). Transformants were firstly screened on selective complete medium (CM) containing 0.5μM 5-fluoro-2'-deoxyuridine (F2dU) and 200μg/ml hygromycin B. Random insertion transformants containing an *HSVtk* gene from pKO3A cannot grow on the F2dU medium. Null mutants were then identified using a double-negative PCR for *PoRAL2* with primer set S-F/S-R, using *β*-*TUBULIN* as a positive control with primer set Tbl-gF/Tbl-gR. Successful recombination of *HPH* into the deleted-*PoRAL2* site was confirmed by another PCR reaction using primer set L-F/*HPH*-CKR. Finally, the inserted copies of *HPH* in the genomic DNA of null mutants were determined by quantitative Real Time PCR (qPCR), using *β-TUBULIN* as a control (primer sets q*HPH*-F/q*HPH*-R and qtubF/qtubR; Lu et al., 2014; Cao et al., 2016). The primers used in this study are listed in Supplementary Table S1.

For complementation experiments, a 6.3kb DNA fragment of *PoRAL2* with native promoter, coding sequence (CDS), and native terminator was PCR amplified from wild-type *P. oryzae* genomic DNA using primer set *PoRAL2*c-F/*PoRAL2*c-R and inserted into an *Eco*RI and *SaI*I-linearized vector pKD5, containing a sulfonylurea resistance gene (*SUR*; Li et al., 2012b). The *PoRAL2* DNA fragment was transformed into ∆*PoRal2 via* ATMT to create the complemented *Poral2c* strain, and the transformants were screened on selection medium containing 100μg/ml sulfonylurea. The mRNA expression level of *PoRAL2* in the complementation strain was confirmed by RT-PCR.

For active *RAS2*^G18V^ allele overexpression (GFP-Ras2^G18V^), the CDS of *RAS2*^G18V^ was fused to the C-terminus of GFP by cloning into *Xba*I digested pKD3-GFP which was built by replacing *SUR* with *BAR* in pKD5-GFP (Li et al., 2012b) and expressed under the control of the H3 promoter. For effector secretion observation (Bas4-mCherry and Pwl2-mCherry-NLS), the native promotor sequence along with the CDSs of *BAS4* and *PWL2* was cloned and fused, respectively, into pKD3-mCherry, constructed by replacing GFP with mCherry in pKD3-GFP, and pKD5-mCherry, constructed by replacing GFP with mCherry in pKD5-GFP containing a nuclear localization signal (NLS) signal at the C-terminus of mCherry. Each of the three cassettes (GFP-Ras2^G18V^, Bas4-mCherry, and Pwl2-mCherry-NLS built in pKD3-GFP-Ras2^G18V^, pKD3-Bas4-mCherry, and pKD5-Pwl2-mCherry-NLS, respectively) was separately transformed into the wild-type and ∆*Poral2* mutant strains. Positive transformants GFP-Ras2^G18V^ were visually confirmed by GFP fluorescence, and those of Bas4-mCherry (Wei et al., 2020) and Pwl2-mCherry-NLS (Khang et al., 2010) by mCherry fluorescence under a fluorescence microscope.

### Mutant Phenotypic Assays

Wild-type, ∆*Poral2*, and *Poral2c P. oryzae* strains were cultured on 7cm plates with 17.5ml CM at 25°C for 10days. For sporulation, conidia were scraped from culture plates, suspended in 3ml of double distilled water (ddH_2_O), and counted with a counting chamber. For conidiophore development, media containing vegetative hyphae were sliced into slender pieces and re-cultured under continuous light, at 25°C for 24h (Cao et al., 2016).

For stress tests, each of the strains was cultured on minimal medium [MM, 10g/L D-Glucose, 6g/L NaNO_3_, 1.52g/L KH_2_PO_4_, 0.52g/L KCl, 0.52g/L MgSO_4_∙7H_2_O, 0.1% (v/v) trace elements, 0.1% (v/v) vitamin solution] with 0.8M sucrose, 0.8M sorbitol, 0.5M NaCl, 20μg/ml Congo red, or 50μg/ml Calcofluor white (CFW) at 25°C (Qu et al., 2020). To assay conidial germination and appressorium formation, 20μl spore suspensions (5×10^4^conidia/ml) were dropped onto hydrophobic coverslips and incubated at 22°C for 4h post-inoculation (hpi; conidial germination) and for 8 and 24hpi (appressorium formation; Shi et al., 2018). To observe cAMP’s role on appressorium formation, 10mM 8-bromoadenosine 3',5'-cyclic monophosphate sodium salt (8-Br-cAMP; Sigma, Japan) was added to spore suspensions which were dropped on hydrophobic or hydrophilic surfaces (GelBond) for 8 and 24hpi. In cytorrhysis (cell collapse) assays (Howard et al., 1991), collapsed cells of 24hpi appressoria were counted after exposure to 1.0, 2.0, 3.0, 5.0, or 6.0M glycerol solutions for 5min. To visualize glycogen production, cells were stained with an iodine solution containing 60mg/ml KI and 10mg/ml I_2_ for 1min (Thines et al., 2000). To stain lipid droplets, conidia were co-cultured with 10μg/ml tricyclazole during appressorium formation, and the fluorescent dye Boron dipyrromethene (BODIPY; Thermo Fisher, United States) was applied to appressoria for 3min (Wang et al., 2018).

### Virulence Test and Infection Process Observation

To detect the influence of *PoRAL2* deletion on virulence, 5×10^4^conidia/ml in 0.2% (w/v) gelatin was sprayed onto 14-day-old rice seedlings (*Oryza sativa* cultivar CO39) and cultured for 2days in darkness at 22°C and 4days under a 16:8-h light/dark cycle at 25°C. Disease lesion severity (disease score) was assessed in a 5-cm-length section of the leaf exhibiting the most serious disease lesions in each seedling (Cao et al., 2016). For penetration assays, 20μl of spore suspensions (5×10^4^conidia/ml) of wild-type, ∆*Poral2*, and *PoRal2c* strains was dropped onto 7-day-old barley leaves (*Hordeum vulgare*) kept at 25°C. Leaves were then collected at 24, 48, 72, or 96hpi, then decolored by methanol, fixed in alcoholic lactophenol, and observed under a microscope (Lu et al., 2007).

### Glycerol Concentration Assay

Conidia scraped from 14-day-old fungal cultures were diluted to 9 ×10^5^conidia/ml in 320μl of conidium solution and dropped onto a hydrophobic surface for 24h. The conidia were scraped *via* eraser and dissolved into 1ml of ddH_2_O, after which the glycerol concentration was tested using the glycerin content GPO-POD enzymatic assay kit (Applygene, China).

### Quantitative Real-Time PCR

For RNA isolation, 200μl of a 5×10^4^conidia/ml spore suspension was spread onto cellophane membranes over CM plates and cultured for 3days. Total RNA was extracted with Trizol, following the manufacturer’s procedure (TaKaRa, Japan), and transcribed into cDNA using the PrimeScriptTM RT reagent kit with gDNA Eraser (TaKaRa, Japan). The qPCR assay was performed on the Real-Time PCR Detection System MasterCycler (Eppendorf, Germany) with the TB Green® Premix Ex Taq™ (Tli RNaseH Plus) kit (TaKaRa, Japan) following the manufacturer’s protocol. Relative abundance of transcripts was assessed by the 2^−∆CT^ method, where ∆CT=CT_gene_−(CT*_40S_*+CT*_ACTIN_*)/2. 2^−∆∆CT^ was used as the standard for calculating fold changes between two strains, where ∆∆CT=∆CT_strain_1_−∆CT_strain_2_ (Livak and Schmittgen, 2001). Tukey’s HSD test was used to assess significance for all experimental data between samples (Tang and Zhang, 2013).

### Yeast Two-Hybrid Assay

The Matchmaker Gal4 Two-Hybrid System 3 (Clontech, United States) was used to assay protein–protein interactions. The cDNA of *PoRAL2*, *GEF1* *SCD1* (MGG_09697), *SMO1* (MGG_03846), *RAS1* (MGG_09499), *RAS2* (MGG_06154), dominant active *RAS2*^G18V^ allele, *CDC42* (MGG_00466), *MST50* (MGG_05199), (MGG_00466), *MST7* (MGG_00800), *PMK1* (MGG_09565), *MST11* (MGG_14847), the half N-terminal fragment (1–1,605bp, *PoRAL2N*), the half C-terminal fragment (1,606–3,210bp, *PoRAL2C*) and the kelch domain (754–1,353bp, *PoRAL2K*) of PoRAL2 cDNA, and three truncated segments of Gef1 cDNA [*GEF1*a (1–2,433bp), *GEF1*b (2434–4,395bp), and *GEF1*c (4,366–6,018bp)], were cloned from a 70–15 strain cDNA library with primers listed in Supplementary Table S1 and ligated into a prey vector, pGADT7, or a bait vector, pGBKT7, with a one-step cloning kit (Vazyme Biotech Co. China). Pairwise combinations of prey and bait constructs, following verification by sequencing, were co-transformed into the yeast strain Y2HGold. The resulting yeast cells, grown on the auxotrophic medium SD-Leu-Trp, were diluted to 1 × 10^6^cells/ml and dropped on SD-Leu-Trp-Ade-His medium for growth. One hundred microliters of X-α-Gal (4mg/ml) was spread onto 9-cm-diameter SD-Leu-Trp-Ade-His plates when testing the interactions between PoRal2 and Mst50, Scd1, Smo1, and Gef1.

### Determination of Non-phosphorylated and Phosphorylated Pmk1 Levels

Total proteins were isolated from 2-day-old vegetative hyphae grown in 5 x YEG culture (5g yeast extract, 10g glucose, 1L ddH_2_O) using TCA-acetone precipitation methods, and total protein concentrations were determined using the Enhanced BCA Protein Assay Kit (Beyotime, China). Protein extracts (50μg) were subjected to 12% SDS-PAGE (EpiZyme, China) and transferred to a PVDF membrane as described in previous research (Zhang et al., 2018). Primary antibodies, including anti-Phospho-p44/42 MAPK antibody #4370 (Cell Signaling Technology, Inc.), anti-ERK1/2 MAPK antibody (C-9): sc-514,302 (Santa Cruz Biotechnology, Inc.), and anti-GAPDH R1208-3 antibody (HUABIO, China), along with peroxidase (HRP)-conjugated goat anti-rabbit and goat anti-mouse secondary antibodies (Beyotime, China), were used in this study. An ECL chemiluminescent kit (Bio-Rad, United States) was used for Western blot detection.

### Co-immunoprecipitation and Pull-Down Assays

For co-immunoprecipitation (CoIP), the N-terminal half DNA fragment of *PoRAL2* (*PoRAL2N*) was cloned and fused to pKD3-GFP, and DNA fragments of *SCD1*, *SMO1*, *MST50* and *GEF1* were cloned into pKD7-3×Flag. The resulting fragments 3×Flag-Scd1, Smo1-3×Flag, Mst50-3×Flag, and 3×Flag-Gef1 were co-transformed together with PoRal2N-GFP into ∆*Poral2 via* ATMT. PoRal2N-GFP and Mst50-3×Flag were precipitated with Anti-GFP Affinity Beads 4FF SA070005 (SMART LIFESCIENCES, China). Proteins were detected by an anti-Flag antibody M1403-2 (HUABIO, China) or an anti-GFP antibody G1544-100UG (Sigma, Japan), respectively.

For pull-down assays, the cDNA fragment of *PoRAL2* was inserted into pET21 with 3×Flag tag, and cDNA of *SCD1*, *SMO1*, *MST50*, *GEF1*, and *PMK1* was inserted into pGEX-4T with GST tag and co-transformed into *Escherichia coli* BL21. Flag-PoRal2 (115.05kDa), GST-Scd1 (141.82kDa), GST-Smo1 (141.82kDa), GST-Mst50 (80.34kDa), GST-Gef1 (246.87kDa), and GST-Pmk1 (68.28kDa) were expressed by induction using 0.2M IPTG for 16h and pulled down by GST beads C600913 (BBI, China). Proteins were detected by an anti-GST antibody EM80701 (HUABIO, China) and an anti-Flag antibody M1403-2 (HUABIO, China), respectively.

## Results

### PoRal2 Is a Kelch Domain Protein

**Figure 1:**
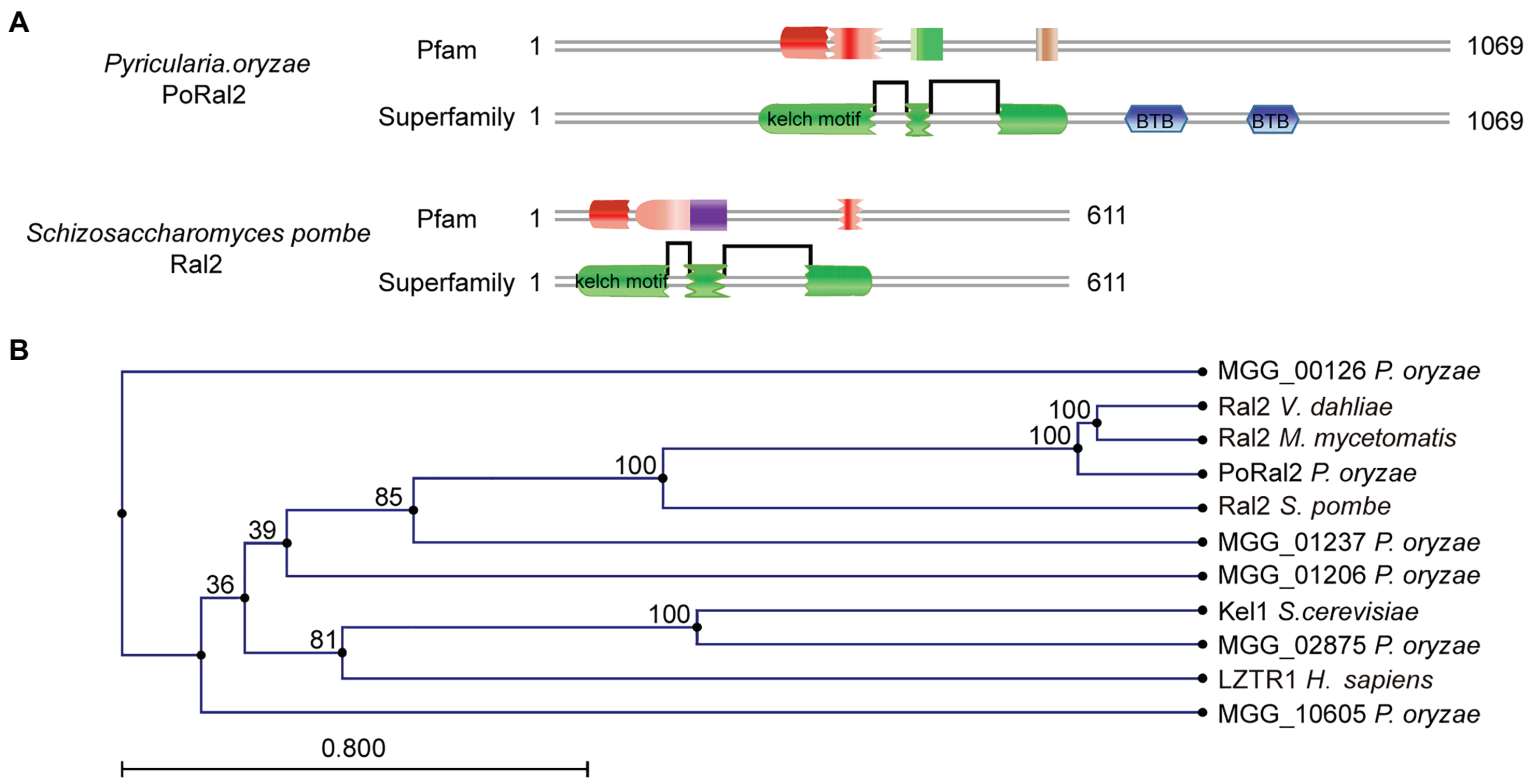
Identification of PoRal2 in *Pyricularia oryzae*. **(A)** Protein sequence analysis *via* the profile-HMM database showed that both *P. oryzae* PoRal2 and *Schizosaccharomyces pombe* Ral2 proteins contain three kelch repeats. **(B)** Alignment tree of PoRal2 and Ral2 proteins from *S. pombe*, *Verticillium dahlia* (XP_009657748), *Madurella mycetomatis* (KXX79671), *Saccharomyces cerevisiae* (Kel1), human (LZTR1), and six kelch domain containing proteins (PoRal2, MGG_00126, MGG_01206, MGG_01237, MGG_02875, and MGG_10605) in *P. oryzae* were constructed using the CLC Main Workbench.

Six kelch domain-containing proteins in *P. oryzae*, including MGG_00126, MGG_01206, MGG_01237, MGG_02875, MGG_08255, and MGG_10605, were identified after BLASTing the *P. oryzae* genome database against Kel1 protein sequences from *S. cerevisiae* (Philips and Herskowitz, 1998). Protein sequence analysis *via* the profile-HMM database^1^ showed that MGG_08255 (named PoRal2) has three kelch domains close to the N-terminal end (Figure 1A). Pfam^2^ and SMART^3^ analysis also showed that PoRal2 has a BTB domain at its C-terminal end. PoRal2 is 1,069 amino acid residues long and shares 29.14% identity with the *S. pombe* Ral2 protein in amino acid sequence. Ral2 is a Ras1-Scd pathway protein and contains three kelch repeats at its N-terminal end in *S. pombe* (Fukui et al., 1989). Among six kelch-domain-containing proteins in *P. oryzae*, PoRal2 showed highest homology to the Ral2 proteins in *S. pombe*, *Verticillium dahlia* (XP_009657748), and *Madurella mycetomatis* (KXX79671; Figure 1B). Until now, except for *S. pombe*, the roles of Ral2 in fungi, including pathogenic filamentous fungi, have not been revealed. Here, we reported the roles of PoRal2 in fungal development and pathogenicity in *P. oryzae*.

### PoRal2 Is Involved in Sporulation and Appressorium Formation in *P. oryzae*

**Figure 2:**
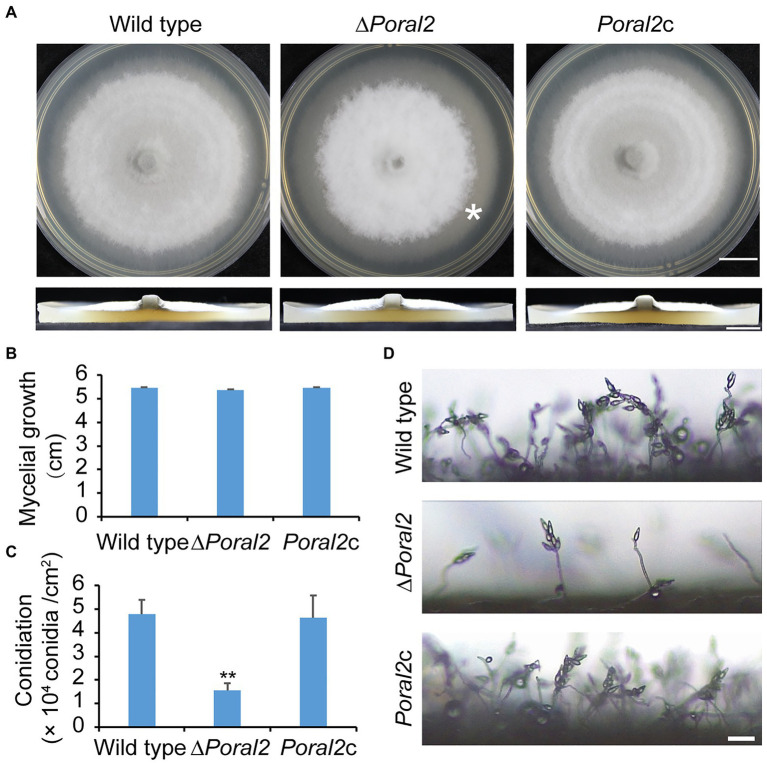
Mycelial growth and sporulation in ∆*Poral2*. **(A)** Colonies of wild type, ∆*Poral2*, and *PoRAL2*-complemented ∆*Poral2* (*Poral2c*) *P. oryzae* strains. Asterisks refer to delays in differentiation of aerial hyphae. The strains were cultured on complete medium (CM) at 25°C for 10days. Bar=1cm. **(B)** Mycelial diameters (cm) of wild-type, ∆*Poral2*, and *Poral2c* colonies. **(C)** Conidiation of wild-type, ∆*Poral2*, and *Poral2c* strains. **(D)** Conidiophore development of *P. oryzae* strains. Bar=20μm. **(B,C)** Error bars represent standard deviations. Significant differences compared with the wild type were estimated by Tukey’s HSD test: ^**^p<0.01.

**Figure 3:**
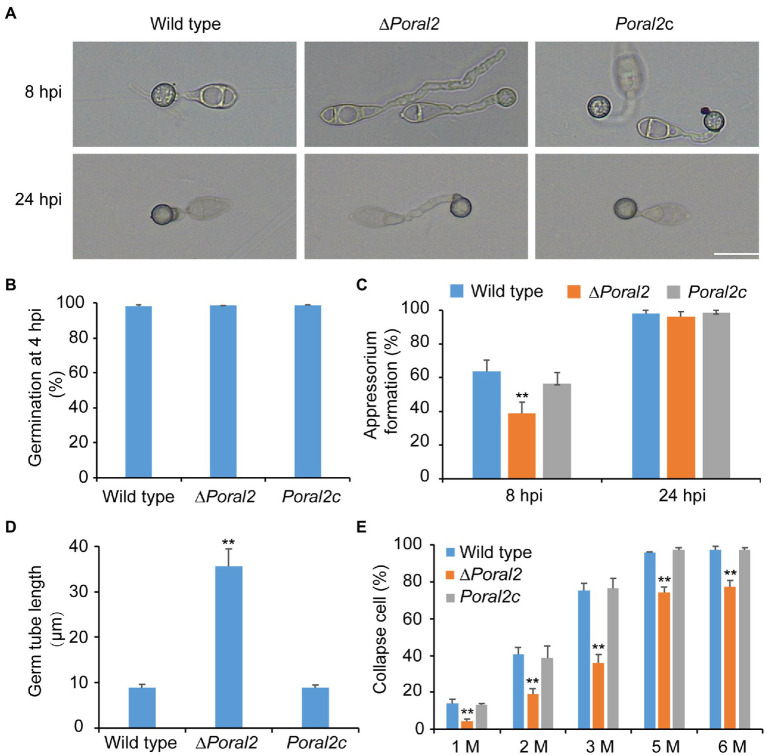
Conidial germination and appressorium formation of wild-type, ∆*Poral2*, and *Poral2c P. oryzae* strains. **(A)** Appressorium formation of wild-type, ∆*Poral2*, and *Poral2c* strains on a hydrophobic surface at 8 and 24hpi. Bar=20μm. **(B)** Conidial germination rate (%) of *P. oryzae* strains at 4hpi. **(C)** Appressorium formation rate (%) of *P. oryzae* strains at 8 and 24hpi. **(D)** Germ tube length (μm) of wild-type, ∆*Poral2*, and *Poral2c* strains. About 100 appressoria were photographed and measured using the software NIS-Elements D 3.2, in triplicate. The ∆*Poral2* strain experienced a 4-fold increase in germ tube length compared to the wild type. **(E)** Collapsed appressoria rates (%) of three *P. oryzae* strains under 1.0, 2.0, 3.0, 5.0, and 6.0M glycerol solutions. The ∆*Poral2* strain showed lower collapse rates under applications of exogenous glycerol solutions compared to the wild type. Error bars represent standard deviations. Significant differences compared with the wild type were estimated by Tukey’s HSD test: ^**^p<0.01.

*PoRAL2* was knocked out in the wild-type *P. oryzae* strain 70–15 (Supplementary Figure S1; Supplementary Table S2) and subsequently complemented with an inserted copy of the native gene (Supplementary Figure S1; Supplementary Table S3). Mycelial growth of the ∆*Poral2* strain was similar to that of the wild type; however, its aerial hyphal differentiation from substrate mycelia was delayed (Figures 2A,B). ∆*Poral2* produced 1.54±0.32×10^4^conidia/cm^2^, significantly less than the wild type (4.78±0.61×10^4^conidia/cm^2^; Figure 2C). Relative to the wild type, ∆*Poral2* produced fewer conidiophores which produced fewer conidia (Figure 2D), indicating decreased sporulation was due to reduced conidiophore differentiation and reduced differentiation of conidia on conidiophores. Spore germination was not affected by *PoRAL2* deletion; however, the appressorial formation rate in ∆*Poral2* was slower than in the wild type at 8hpi, but comparable to the wild type by 24hpi, indicating delayed appressorial formation in the mutant (Figures 3A–C). Noticeably, the germ tubes of ∆*Poral2* mutants were significantly longer than the wild type when germinated on a hydrophobic plastic membrane (Figures 3A,D). The wild-type conidia generated germ tubes about 8.90±0.80μm long, while ∆*Poral2* conidia generated germ tubes about 35.56±3.92μm long. Therefore, we propose that delayed appressorium formation in the mutant is due to more time spent on germ tube elongation.

Appressorium turgor was evaluated according to previous reports (Howard et al., 1991; Lu et al., 2007) by counting the collapse rates of appressoria exposed to 1.0–6.0M glycerol solutions. The collapse rates of ∆*Poral2* appressoria were significantly lower than those of the wild-type and *Poral2c* strains (Figure 3E). At a 3.0M glycerol concentration, 75.2% of the wild-type appressoria had collapsed while only 35.9% of ∆*Poral2* appressoria had collapsed. Lipids and glycogen are two primary nutrient stocks in conidia used to produce intracellular glycerol, which assures the generation of the huge appressorium turgor (de Jong et al., 1997; Foster et al., 2003; Wilson and Talbot, 2009). We examined the translocation and degradation of lipid droplets and glycogen from spores to appressoria at 0, 8, 16, or 24hpi. The wild-type, ∆*Poral2*, and *Poral2c* strains showed similar patterns of lipid droplet and glycogen translocation and degradation during appressorium formation (Supplementary Figure S2), suggesting that lipid and glycogen catabolism were normal in the ∆*Poral2* mutant. We also determined the glycerol concentration in appressoria at 24hpi, and no significant difference was detected between the ∆*Poral2* mutant and the wild type (Supplementary Figure S2).

As the osmotic pathway and cell wall integrity are both involved in maintaining appressorium turgor (Ryder et al., 2019), we tested the growth of the wild-type, ∆*Poral2*, and *Poral2c* strains on MM media supplemented with 0.8M sucrose, 0.8M sorbitol, 0.5M NaCl, 20μg/ml Congo red, or 50μg/ml CFW. Relative growth rates of the ∆*Poral2* strain on the above-mentioned media were comparable to those of the wild-type and *Poral2c* strains (Supplementary Figure S3), and no significant growth defects in the ∆*Poral2* strain was identified under osmotic and cell wall stresses.

### PoRal2 Is Required for Fungal Response to cAMP Signaling and Involved in the Pmk1 MAPK Pathway

**Figure 4:**
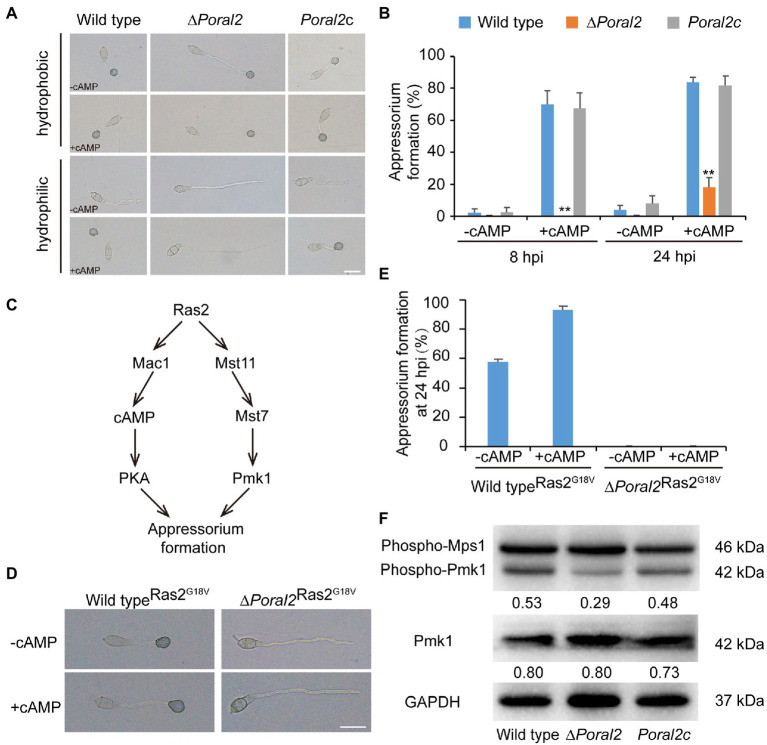
Defects of ∆*Poral2* in the cAMP-PKA and Pmk1 MAPK signaling pathways. **(A)** Appressorium formation of *P. oryzae* strains on hydrophobic or hydrophilic surfaces with or without supplemented 10mM 8-Br-cAMP at 24hpi. Bar=20μm. **(B)** Appressorium formation rate (%) of *P. oryzae* strains on hydrophobic and hydrophilic surfaces with or without supplemented 10mM 8-Br-cAMP at 8 and 24hpi. Conidial suspensions were dropped onto GelBond film and incubated at 22°C. Significant differences compared with the wild type were estimated by Tukey’s HSD test: ^**^p<0.01. **(C)** Regulatory relations between Ras2 and the cAMP-PKA pathway and Pmk1 MAPK pathway. **(D,E)** Appressorium formation of *P. oryzae* strains on hydrophilic surfaces with or without supplemented 8-Br-cAMP when *RAS2*^G18V^ was overexpressed in both the wild-type and *∆Poral2* strains. Bar=20μm. **(F)** Expression levels of Mps1 and Pmk1 kinases was detected by Western blot using anti-Phospho-p44/42 MAPK antibody (top panel), anti-ERK1/ERK2 MAPK antibody (middle panel), and anti-GAPDH antibody (lower panel). Numbers under the pictures represent the protein contents of phospho-Pmk1 or total Pmk1 relative to that of GAPDH (mean, *n* =3 independent experiments). The protein contents were determined according to gray values of Western blot bands measured by Image J. The phosphorylation level of Pmk1 in the ∆*Poral2* mutant was reduced compared to the wild type.

Exogenous cAMP stimulates appressorium formation in wild-type and mutant *P. oryzae* strains harboring defects in the cAMP-PKA (cAMP-dependent protein kinase A) signaling pathway, such as ∆*mgb1* and ∆*mac1* (Choi and Dean, 1997; Nishimura et al., 2003), but not in mutants which displayed defects in the *MST11*-*MST7*-*PMK1* MAPK (mitogen-activated protein kinase) signaling pathway, such as ∆*mst7*, ∆*mst11*, and ∆*pmk1* (Xu and Hamer, 1996; Zhao et al., 2005). Adenosine 3',5'-cyclic monophosphate sodium salt (8-Br-cAMP), a cell-permeable cAMP analog, is a naturally-occurring activator of PKA that contributes to appressorium formation (Li et al., 2019). Appressorium formation of the ∆*Poral2* strain exposed to 10mM 8-Br-cAMP was tested on hydrophobic and hydrophilic surfaces (GelBond, United States). As shown in Figure 4A, the ∆*Poral2* mutant produced long germ tubes on hydrophobic surfaces with or without 8-Br-cAMP. After addition of 8-Br-cAMP, most conidia of both the wild-type and *Poral2c* strains (83.80 and 81.79%, respectively) produced appressoria on hydrophilic surfaces (Figures 4A,B). On the contrary, 82% of the ∆*Poral2* mutant conidia did not produce appressoria and merely generated long and straight germ tubes at 24hpi, whether or not 8-Br-cAMP was present (Figures 4A,B).

The dominant expression of the activated *RAS2*^G18V^ allele causes the wild-type strain to form appressoria on a hydrophilic surface by activating cAMP-PKA and Pmk1 MAPK pathways (Zhou et al., 2014; Figure 4C). We overexpressed the activated *RAS2*^G18V^ allele C-terminally fused to GFP under the control of the *H3* promoter in wild-type and ∆*Poral2* strains. Overexpression of *RAS2*^G18V^ in the wild type permitted appressorium formation on a hydrophilic surface (Figures 4D,E). However, overexpression of *RAS2*^G18V^ in the ∆*Poral2* mutant strain did not permit appressorium formation on a hydrophilic surface, even in the presence of 8-Br-cAMP (Figures 4D,E). The Pmk1 MAPK cascade is another signaling pathway that controls appressorium formation, and deletion of *PMK1* results in the inability to form appressorium (Xu and Hamer, 1996). We determined the levels of non-phosphorylated and phosphorylated Pmk1 proteins *via* Western blot, using anti-ERK1/ERK2 MAPK and anti-Phospho-p44/42 MAPK antibodies, respectively. As shown in Figure 4F, the protein levels of non-phosphorylated Pmk1 in the ∆*Poral2* strain were comparable to the wild type, whereas phosphorylated Pmk1 levels were reduced. In contrast, the Mps1 phosphorylation level of the ∆*Poral2* strain was comparable to the wild type (Figure 4F), implying that Pmk1 phosphorylation, but not Mps1 phosphorylation, is specifically reduced in the ∆*Poral2* strain. The imperfect responses to exogenous cAMP and activated Ras2^G18V^ on a non-inducing surface, and the reduced phosphorylated Pmk1 levels displayed by the ∆*Poral2* strain implied that PoRal2 is involved in both the cAMP-PKA and Pmk1 MAPK signaling pathways.

### Deletion of *PoRAL2* Leads to Down-Regulation of Genes Involved in Signaling Pathways Required for Appressorium Formation

**Figure 5:**
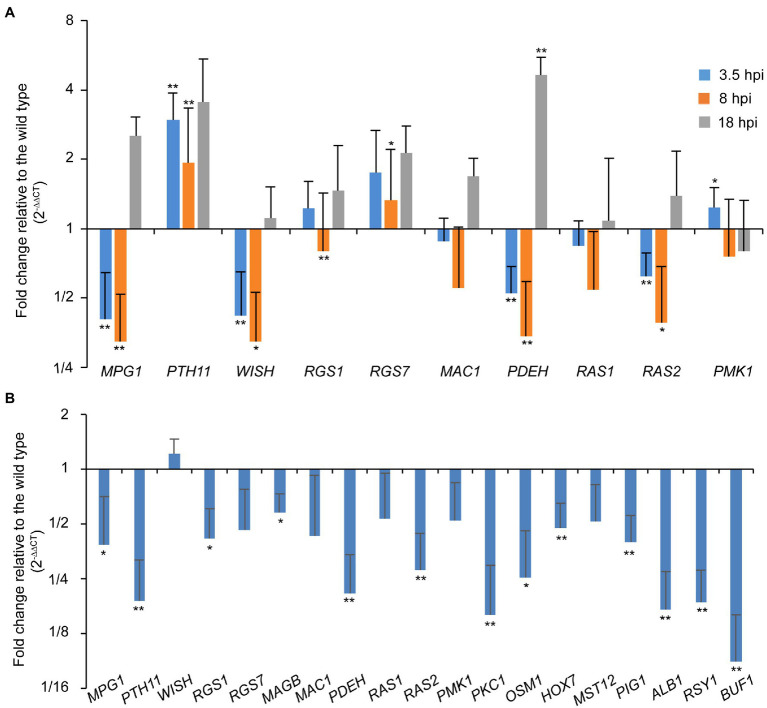
PoRal2 is required for the expression of genes involved in appressorium formation in *P. oryzae*. **(A)** Relative mRNA expression levels in germinating spores at 3.5, 8, and 18hpi of the ∆*Poral2* strain. **(B)** Relative mRNA expression levels in the aerial hyphae in the ∆*Poral2* strain. Transcript levels of *MPG1*, *PTH11*, *WISH*, *RGS1*, *RGS7*, *MAGB*, *MAC1*, *PDEH*, *RAS1*, *RAS2*, *PMK1*, *PKC1*, and *OSM1* genes involved in cAMP signaling and MAP kinase cascade pathways, and *PIG1*, *ALB1*, *BUF1*, and *RSY1* genes involved in melanin synthesis were determined in the wild-type and ∆*Poral2* strains by RT-qPCR. The reference genes used were *ACTIN* and *40S*. Significant differences compared with the wild type were estimated by Tukey’s HSD test: ^*^p<0.05 and ^**^p<0.01.

As the ∆*Poral2* strain did not respond to exogenous cAMP on hydrophilic surfaces in the same manner as the wild type, we measured the relative expression levels of *MPG1*, *PTH11*, *WISH*, *RGS1*, and *RGS7* involved in surface recognition and signal transition (Talbot et al., 1996; Liu and Dean, 1997; DeZwaan et al., 1999; Liu et al., 2007; Kou et al., 2017; Sabnam and Barman, 2017), *MAC1* and *PDEH* in the cAMP-PKA pathway (Choi and Dean, 1997; Ramanujam and Naqvi, 2010), as well as *RAS1*, *RAS2*, and *PMK1* in the Pmk1 MAPK pathway (Zhou et al., 2014) during appressorium formation. At 3.5 and 8hpi, *MPG1*, *WISH*, *PDEH*, and *RAS2* were significantly down-regulated, while *PTH11* was significantly up-regulated. *RGS1* was significantly down-regulated at 8 hpi, while *RGS7* at 8hpi and *PDEH* at 18hpi were significantly up-regulated (Figure 5A). In aerial hyphae, *PKC1* and *OSM1* involved in cell wall integrity and osmotic signaling pathways (Ryder et al., 2019), and *PIG1*, *ALB1*, *RSY1*, and *BUF1* involved in the melanin synthesis pathway (Tsuji et al., 2000; Zhu et al., 2020), and *MPG1*, *PTH11*, *RGS1*, *PDEH*, and *RAS2* were all significantly down-regulated in the ∆*Poral2* mutant (Figure 5B). Down-regulation of *PIG1*, *ALB1*, *RSY1*, and *BUF1* was correlated with the less melanized aerial mycelium of the ∆*Poral2* mutant (Figure 2A). These results suggest that the expression of several pivotal genes in the cAMP-PKA, Pmk1 MAPK, and melanin synthesis was altered by deletion of *PoRAL2* in *P. oryzae*.

### PoRal2 Is Involved in Plant Penetration and Virulence of *P. oryzae*

**Figure 6:**
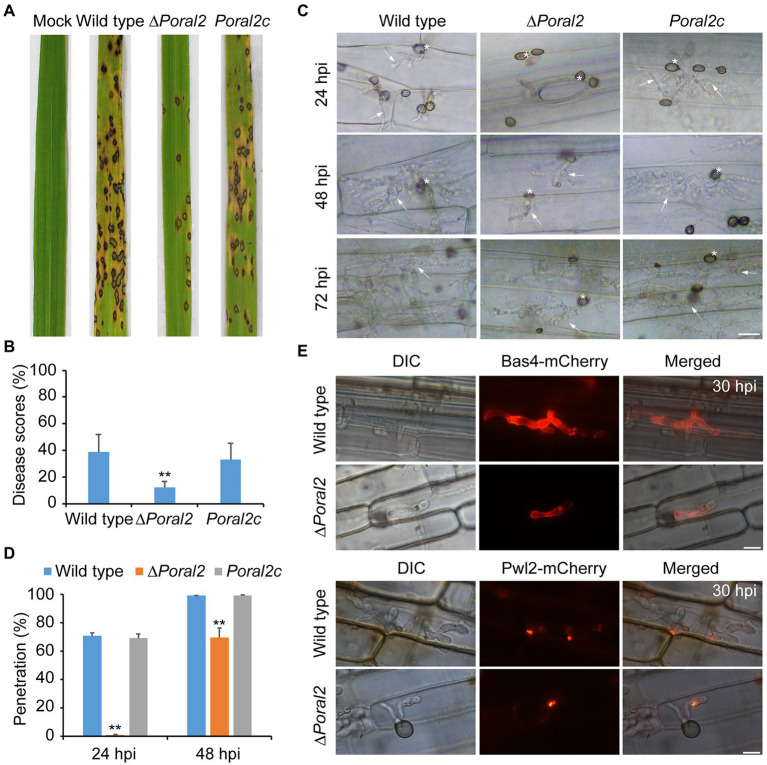
Virulence and plant penetration assays. **(A)** Conidial suspensions (5×10^4^conidia/ml) were sprayed onto 14-day-old rice seedlings and cultured for 2days in darkness at 22°C and 4days under a 16:8-h light/dark cycle at 25°C. **(B)** Disease scores for the wild-type, ∆*Poral2*, and *Poral2c* strains were measured with 5-cm leaf length by Photoshop CS6. **(C)** Penetration and invasive growth in barley leaves at 24, 48, and 72hpi. Bar=20μm. Asterisks indicate appressoria, and arrows indicate invasive hyphae. **(D)** Penetration rate (%) of *P. oryzae* appressoria at 24 and 48hpi. 20μl of conidial suspensions (5×10^4^conidia/ml) was inoculated onto intact barley leaves at 25°C. **(E)** Location of Bas4-mCherry and Pwl2-mCherry-NLS in wild-type and ∆*Poral2* strains in leaf sheaths at 30hpi. Bar=10μm. **(B,D)** Error bars represent standard deviations. Significant differences compared with the wild type were estimated by Tukey’s HSD test: ^*^p<0.05 and ^**^p<0.01.

After spraying spore suspensions (5×10^4^conidia/ml) on rice seedlings, which were then cultured in the dark at 22°C for 2days and under a 16:8h light/dark cycle at 25°C for 4days, the disease incidence of the ∆*Poral2* strain was only 12.12%, whereas that of the wild type reached 39.02%, suggesting that the virulence of the ∆*Poral2* strain was significantly reduced (Figures 6A,B). To test appressorium penetration ability, conidia were dropped onto 7-day-old barley leaves and cultured at 25°C, and the status of appressorium penetration was checked at 24 and 48hpi. At 24hpi, the ∆*Poral2* mutant had only formed appressoria on barley leaves, while appressoria of the wild-type and *Poral2c* strains had already produced short penetration pegs inside leaf cells (Figure 6C). At 48hpi, while the ∆*Poral2* strain had just begun producing differentiated bulbous hyphae in the first infected cell, hyphae of the wild-type strain had already invaded into neighboring cells. The percentage of appressoria that had penetrated into barley cells was 70.6% for the wild type and 0.9% for ∆*Poral2* at 24hpi, and 98.93 and 69.7% at 48hpi, respectively (Figure 6D). Therefore, penetration into the plant host by the ∆*Poral2* mutant was significantly delayed, suggesting *PoRAL2* is a virulence gene for *P. oryzae*.

During infection, *P. oryzae* secretes effectors at the pathogen–host cell membrane interface to promote biotrophic invasion and overcome host plant defenses (Khang et al., 2010). Pwl2 is a cytoplasmic effector, located in the BIC (biotrophic interfacial complex) at primary invasive hyphal tips, which is translocated into the rice cytoplasm, while Bas4 is an apoplastic effector located in plant-derived EIHMs (extrainvasive hyphal membranes) covering the invasive hyphae (Khang et al., 2010). Given that penetration and invasive growth were delayed in the ∆*Poral2* mutant, we observed the location of Bas4-mCherry and Pwl2-mCherry-NLS after inoculation of the wild-type and ∆*Poral2* strains onto rice sheaths for 30h. Secretion of neither Bas4 nor Pwl2 by ∆*Poral2* showed apparent differences from secretion of these proteins by the wild-type strain, although infection by the ∆*Poral2* strain was delayed in rice sheath cells (Figure 6E).

### PoRal2 Protein-Interaction Assays

**Figure 7:**
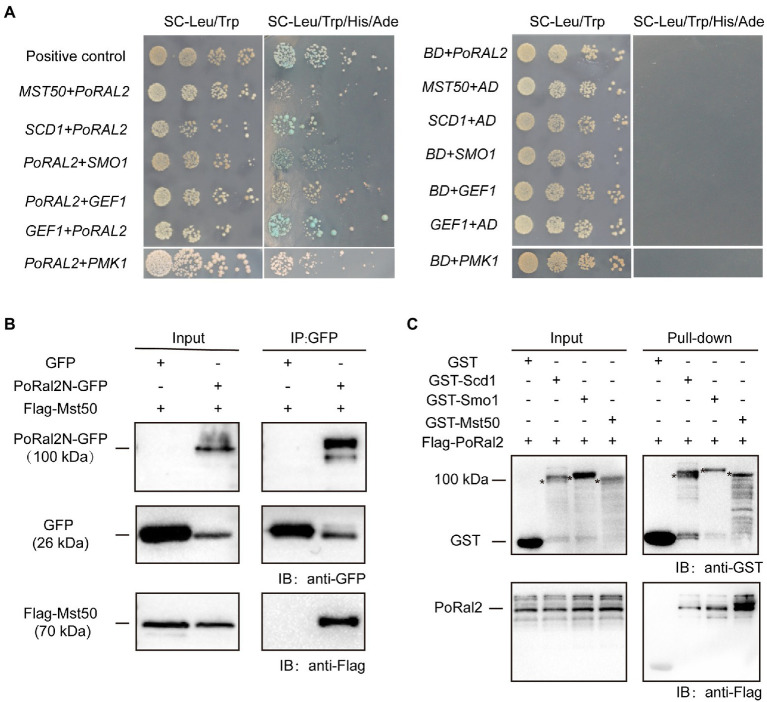
Protein interactions assays. **(A)** Yeast Two-Hybrid Assays. PoRal2 and Smo1, Pmk1, Gef1, Scd1 or Mst50 cloned in pGADT7 or pGBKT7 were co-transformed into yeast and cultured on selection media. **(B)** Co-immunoprecipitation results of PoRal2N-GFP and Mst50-3×Flag in *P. oryzae*. PoRal2N-GFP and Mst50-3×Flag was detected in PoRal2N-GFP elution buffer, while only GFP was detected in GFP elution buffer. **(C)** Pull-down results of 3×Flag-PoRal2 between GST, GST-Scd1, GST-Smo1, and GST-Mst50 in *E. coli* BL21. 3×Flag-PoRal2 was detected in GST-Scd1, GST-Smo1 and GST-Mst50, but not in GST alone elution buffer. Asterisks represented the band of Scd1 (141.82kDa), Smo1 (141.82kDa), or Mst50 (80.34kDa), respectively.

In *S. pombe*, Ral2 is a Ras1-Scd pathway protein which physically interacts with Gef1 (Fukui et al., 1989; Tafforeau et al., 2006). We found that overexpression of *SCD1* in ∆*Poral2* led to generation of longer germ tube in the mutant (Supplementary Figure S4). In *P. oryzae*, Ras1 and Ras2 interact with Gef1, Smo1, Mst11, and Mst50 (Park et al., 2006; Kershaw et al., 2019). We identified the interactions between PoRal2 and several proteins in the Pmk1 MAPK signaling pathway using the yeast two hybrid systems (Y2H). PoRal2 interacted with Mst50, Scd1, Smo1, Gef1, and Pmk1 in Y2H (Figure 7A). Mst50 interacted with Cdc42, Gef1, and Smo1 in Y2H (Supplementary Figure S5). Furthermore, PoRal2N or PoRal2K, but not PoRal2C strongly interacted with Scd1, Mst50, and Gef1; none of PoRal2N, PoRal2K, or PoRal2C interacted with Smo1 in Y2H (Supplementary Figure S6). Whether Gef1 was used as the prey or a bait protein, it always interacted with PoRal2 in Y2H; and Gef1a and Gef1c interacted with PoRal2N in Y2H (Figure 7A; Supplementary Figure S6). To confirm the PoRal2-interacted proteins revealed by Y2H, CoIP, and/or pull-down assays were conducted. In CoIP experiments, Mst50 could be precipitated by PoRal2N (Figure 7B). In pull-down experiments, Scd1, Smo1, and Mst50 were pulled down by PoRal2 (Figure 7C). Thus, PoRal2 physically interacted with Mst50, Scd1, and Smo1 in *P. oryzae*.

## Discussion

The kelch superfamily is one of the largest evolutionarily conserved protein families in metazoan organisms, which are classified by the types and numbers of kelch-repeat, BTB/POZ, and BACK domains (Prag and Adams, 2003). Kelch-repeat proteins are known to control multiple cellular processes, such as actin dynamics, cell morphology maintenance, the cell cycle, oxidative stress responses, and gene expression regulation (Adams et al., 2000). In this study, we identified and characterized the N-terminal kelch repeat and C-terminal BTB domain containing protein, PoRal2, in *P. oryzae* (Figure 1A), and found that PoRal2 plays vital roles in aerial hyphal differentiation, conidiophore and spore development, appressorium formation, plant penetration, and fungal virulence (Figures 2–6).

**Figure 8:**
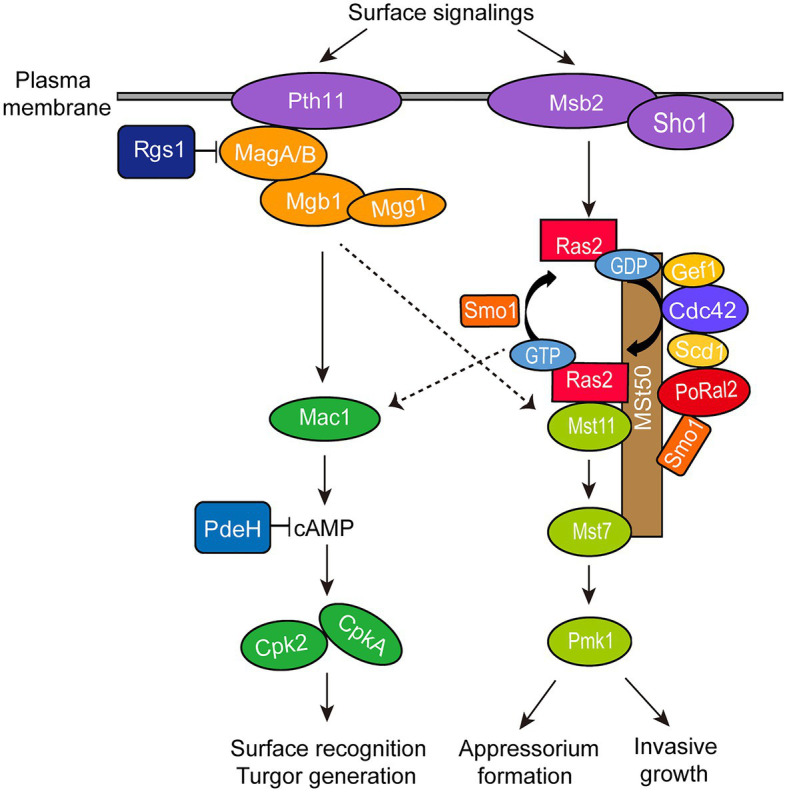
Model for PoRal2 roles during surface signaling transduction and appressorium formation in *P. oryzae*. PoRal2 interacts with Smo1, Scd1, and Mst50 to regulate the activation of the Mst11-Mst7-Pmk1 MAPK pathway by Ras2, thereby controlling the appressorium formation and invasive growth process.

∆*Poral2* displayed longer germ tubes, delayed appressorium formation, and decreased infection growth and virulence (Figures 4A,B, 6). These mutant phenotypic characteristics might be caused by its defects in the cAMP-PKA and Pmk1 MAPK pathways (Figure 4). Similar phenotypes were observed in the mutants that were defective in the cAMP-PKA or Pmk1 MAPK pathways. ∆*cpka* in which one of the two PKA catalytic subunit genes was deleted showed longer germ tubes, delayed appressorium formation, as well as loss of virulence (Mitchell and Dean, 1995; Selvaraj et al., 2017). ∆*magB*, ∆*mgb1*, and ∆*mac1* that were defective in the cAMP-PKA pathway showed longer germ tubes and reduced virulence (Choi and Dean, 1997; Liu and Dean, 1997; Nishimura et al., 2003). Blocking the Pmk1 MAPK pathway, such as in ∆*mst11*, ∆*mst7*, and ∆*pmk1*, led to longer germ tubes that formed subapical swollen bodies but failed to differentiate into appressoria and lose virulence (Zhao et al., 2005; Zhao and Xu, 2007; Qi et al., 2015). Addition of exogenous cAMP or expression of activated *RAS2*^G18V^ could not help ∆*pmk1* develop appressoria (Xu and Hamer, 1996; Zhou et al., 2014). In this study, ∆*Poral2* also failed to respond to exogenous cAMP or expression of activated *RAS2*^G18V^ on hydrophilic surface and showed decreased Pmk1 phosphorylation levels (Figures 4D–F). In addition to appressorium formation, Pmk1 is involved in plant penetration. Strain *pmk1^AS^*, a complemented strain of ∆*pmk1* by an analog-sensitive (AS) allele of *PMK1*, formed appressoria normally in the presence of 5μM 1NA-PP1 (an ATP analog 1-naphthyl-PP1) but displayed reduced penetration ability and invasive growth (Sakulkoo et al., 2018). Furthermore, Pmk1 regulated the transcriptional factor *MST12* which particularly controlled plant penetration (Park et al., 2002). Therefore, we postulated that the deletion of *PoRAL2* led to reduced Pmk1 activation that is responsible for reduced plant penetration. In *S. pombe*, Ral2 is a Ras1-Scd pathway protein found to physically interact with Gef1 (Tafforeau et al., 2006; Vo et al., 2016). Gef1 is a GMP exchange factor (GEF) of Cdc42 (Vo et al., 2016). In *P. oryzae*, Ras1 and Ras2 have been reported to physically interact with the GEF, Gef1, the GTPase-activating protein, Smo1, the MEK kinase, Mst11, and the scaffold protein, Mst50, which interacts with Mst11, Mst7 and Cdc42 (Park et al., 2006; Kershaw et al., 2019). In this study, PoRal2 was found to physically interact with Mst50, Scd1, and Smo1. The interaction between PoRal2 and Gef1 was also confirmed by Y2H. It was found that the N-terminal half fragment (PoRal2N) or kelch domain (PoRal2K) of PoRal2 was enough for its interaction with Scd1, Gef1 and Mst50; Gef1a and Gef1c were enough for Gef1 to interact with PoRal2N in Y2H (Supplementary Figure S6). Considering that Ral2 possibly functions as a Ras-like protein which works closely to Ras1 in *S. pombe* (Philips and Herskowitz, 1998), and PoRal2 from *P. oryzae* interacted with most of the components reported to interact with Ras1 and Ras2 in the upstream of the Pmk1 MAPK pathway, like Mst50, Smo1, Gef1, and Scd1 (Figure 7; Supplementary Figure S5), we proposed that PoRal2 may connect with Mst50, Scd1, Gef1, and Smo1 and function upstream of the Pmk1 MAPK pathway (Figure 8). Mst50 is an adapter protein involved in the Pmk1 MAPK, Mps1 MAPK, and Osm1 MAPK pathways (Li et al., 2017), suggesting that PoRal2 possibly plays its physiological functions in appressorium formation and infection *via* the Mps1 MAPK and Osm1 MAPK pathways.

In summary, we characterized the biological functions of *P. oryzae* PoRal2, a kelch and BTB domain protein homologous to the *S. pombe* Ral2 protein. PoRal2 is involved in aerial hypha and spore differentiation, germ tube extension, appressorium turgor generation, plant penetration, invasive growth, and virulence in *P. oryzae*. PoRal2 plays roles in the cAMP-PKA signaling pathway and functions upstream of the Pmk1 MAPK pathway through interaction with Scd1, Smo1, and Mst50 proteins.

## Data Availability Statement

The original contributions presented in the study are included in the article/Supplementary Material, further inquiries can be directed to the corresponding authors.

## Author Contributions

YQ and JL contributed to the experimental design. YQ, JW, and PH contributed to the experiments. YQ, JW, and JL contributed to the data analysis and scripts. FL, XL, and JL supplied experimental conditions. YQ, JL, FL, and XL wrote the manuscript. All authors contributed to the article and approved the submitted version.

## Funding

This research was supported by the National Natural Science Foundation of China (Grant Nos: 31871908 and 31671975) and Key R&D projects of Zhejiang Province (Grand No: 2021C02010).

## Conflict of Interest

The authors declare that the research was conducted in the absence of any commercial or financial relationships that could be construed as a potential conflict of interest.

## Publisher’s Note

All claims expressed in this article are solely those of the authors and do not necessarily represent those of their affiliated organizations, or those of the publisher, the editors and the reviewers. Any product that may be evaluated in this article, or claim that may be made by its manufacturer, is not guaranteed or endorsed by the publisher.

## Footnotes

^1^ https://www.ebi.ac.uk/Tools/hmmer/search/hmmscan

^2^ https://pfam.xfam.org/search/sequence

^3^ http://smart.embl-heidelberg.de

## References

[ref1] Adams J. Kelso R. Cooley L. (2000). The kelch repeat superfamily of proteins: propellers of cell function. Trends Cell Biol. 10, 17–24. doi: 10.1016/S0962-8924(99)01673-6, PMID: 10603472

[ref2] Belotti F. Tisi R. Paiardi C. Rigamonti M. Groppi S. Martegani E. (2012). Localization of Ras signaling complex in budding yeast. BBA-Mol. Cell Res. 1823, 1208–1216. doi: 10.1016/j.bbamcr.2012.04.016, PMID: 22575457

[ref3] Cao H. J. Huang P. Y. Zhang L. L. Shi Y. K. Sun D. D. Yan Y. X.. (2016). Characterization of 47 Cys_2_-His_2_ zinc finger proteins required for the development and pathogenicity of the rice blast fungus *Magnaporthe oryzae*. New Phytol. 211, 1035–1051. doi: 10.1111/nph.13948, PMID: 27041000

[ref4] Choi W. B. Dean R. A. (1997). The adenylate cyclase gene *MAC1* of *Magnaporthe grisea* controls appressorium formation and other aspects of growth and development. Plant Cell 9, 1973–1983. doi: 10.1105/tpc.9.11.1973, PMID: 9401122 PMC157051

[ref5] de Jong J. C. McCormack B. J. Smirnoff N. Talbot N. J. (1997). Glycerol generates turgor in rice blast. Nature 389, 244–245. doi: 10.1038/38418

[ref6] Dezwaan T. M. Carroll A. M. Valent B. Sweigard J. A. (1999). *Magnaporthe grisea* Pth11p is a novel plasma membrane protein that mediates appressorium differentiation in response to inductive substrate cues. Plant Cell 11, 2013–2030. doi: 10.1105/tpc.11.10.2013, PMID: 10521529 PMC144101

[ref7] Ebbole D. J. (2007). *Magnaporthe* as a model for understanding host-pathogen interactions. Annu. Rev. Phytopathol. 45, 437–456. doi: 10.1146/annurev.phyto.45.062806.094346, PMID: 17489691

[ref8] Foster A. J. Jenkinson J. M. Talbot N. J. (2003). Trehalose synthesis and metabolism are required at different stages of plant infection by *Magnaporthe grisea*. EMBO J. 22, 225–235. doi: 10.1093/emboj/cdg018, PMID: 12514128 PMC140093

[ref9] Fukui Y. Miyake S. Satoh M. Yamamoto M. (1989). Characterization of the *Schizosaccharomyces pombe ral2* gene implicated in activation of the *ras1* gene product. Mol. Cell. Biol. 9, 5617–5622. doi: 10.1128/mcb.9.12.5617-5622.1989, PMID: 2586528 PMC363732

[ref10] Howard R. J. Ferrari M. A. Roach D. H. Money N. P. (1991). Penetration of hard substrates by a fungus employing enormous turgor pressures. Proc. Natl. Acad. Sci. U. S. A. 88, 11281–11284. doi: 10.1073/pnas.88.24.11281, PMID: 1837147 PMC53118

[ref11] Kankanala P. Czymmek K. Valent B. (2007). Roles for rice membrane dynamics and plasmodesmata during biotrophic invasion by the blast fungus. Plant Cell 19, 706–724. doi: 10.1105/tpc.106.046300, PMID: 17322409 PMC1867340

[ref12] Kershaw M. J. Basiewicz M. Soanes D. M. Yan X. Ryder L. S. Csukai M.. (2019). Conidial morphogenesis and septin-mediated plant infection require Smo1, a Ras GTPase-activating protein in *Magnaporthe oryzae*. Genetics 211, 151–167. doi: 10.1534/genetics.118.301490, PMID: 30446520 PMC6325701

[ref13] Khang C. H. Berruyer R. Giraldo M. C. Kankanala P. Park S. Y. Czymmek K.. (2010). Translocation of *Magnaporthe oryzae* effectors into rice cells and their subsequent cell-to-cell movement. Plant Cell 22, 1388–1403. doi: 10.1105/tpc.109.069666, PMID: 20435900 PMC2879738

[ref14] Kou Y. J. Naqvi N. I. (2016). Surface sensing and signaling networks in plant pathogenic fungi. Semin. Cell Dev. Biol. 57, 84–92. doi: 10.1016/j.semcdb.2016.04.019, PMID: 27133541

[ref15] Kou Y. J. Tan Y. H. Ramanujam R. Naqvi N. I. (2017). Structure-function analyses of the Pth11 receptor reveal an important role for CFEM motif and redox regulation in rice blast. New Phytol. 214, 330–342. doi: 10.1111/nph.14347, PMID: 27898176

[ref16] Li H. J. Lu J. P. Liu X. H. Zhang L. L. Lin F. C. (2012b). Vectors building and usage for gene knockout, protein expression and fluorescent fusion protein in the rice blast fungus. J. Agric. Biotech. 20, 94–104.

[ref17] Li G. T. Zhang X. Tian H. Choi Y. E. Tao W. A. Xu J. R. (2017). *MST50* is involved in multiple MAP kinase signaling pathways in *Magnaporthe oryzae*. Environ. Microbiol. 19, 1959–1974. doi: 10.1111/1462-2920.13710, PMID: 28244240

[ref18] Li X. Zhong K. L. Yin Z. Y. Hu J. X. Wang W. H. Li L. W.. (2019). The seven transmembrane domain protein MoRgs7 functions in surface perception and undergoes coronin MoCrn1-dependent endocytosis in complex with G subunit MoMagA to promote cAMP signaling and appressorium formation in *Magnaporthe oryzae*. PLoS Pathog. 15:e1007382. doi: 10.1371/journal.ppat.1007382, PMID: 30802274 PMC6405168

[ref19] Li G. T. Zhou X. Y. Xu J. R. (2012a). Genetic control of infection-related development in *Magnaporthe oryzae*. Curr. Opin. Microbiol. 15, 678–684. doi: 10.1016/j.mib.2012.09.004, PMID: 23085322

[ref20] Liu S. H. Dean R. A. (1997). G protein α subunit genes control growth, development, and pathogenicity of *Magnaporthe grisea*. Mol. Plant Microbe Interact. 10, 1075–1086. doi: 10.1094/MPMI.1997.10.9.1075, PMID: 9390422

[ref21] Liu H. Suresh A. Willard F. S. Siderovski D. P. Lu S. Naqvi N. I. (2007). Rgs1 regulates multiple Gα subunits in *Magnaporthe* pathogenesis, asexual growth and thigmotropism. EMBO J. 26, 690–700. doi: 10.1038/sj.emboj.7601536, PMID: 17255942 PMC1794393

[ref22] Livak K. J. Schmittgen T. D. (2001). Analysis of relative gene expression data using real-time quantitative PCR and the 2^-∆∆CT^ method. Methods 25, 402–408. doi: 10.1006/meth.2001.1262, PMID: 11846609

[ref23] Lu J. P. Cao H. J. Zhang L. L. Huang P. Y. Lin F. C. (2014). Systematic analysis of Zn_2_Cys2 transcription factors required for development and pathogenicity by high-throughput gene knockout in the rice blast fungus. PLoS Pathog. 10:e1004432. doi: 10.1371/journal.ppat.1004432, PMID: 25299517 PMC4192604

[ref24] Lu J. P. Feng X. X. Liu X. H. Lu Q. Wang H. K. Lin F. C. (2007). Mnh6, a nonhistone protein, is required for fungal development and pathogenicity of *Magnaporthe grisea*. Fungal Genet. Biol. 44, 819–829. doi: 10.1016/j.fgb.2007.06.003, PMID: 17644013

[ref25] McDonough K. A. Rodriguez A. (2012). The myriad roles of cyclic AMP in microbial pathogens: from signal to sword. Nat. Rev. Microbiol. 10, 27–38. doi: 10.1038/nrmicro2688, PMID: 22080930 PMC3785115

[ref26] Milburn M. V. Tong L. Devos A. M. Brunger A. Yamaizumi Z. Nishimura S.. (1990). Molecular switch for signal transduction: structural differences between active and inactive forms of protooncogenic ras proteins. Science 247, 939–945. doi: 10.1126/science.2406906, PMID: 2406906

[ref27] Mitchell T. K. Dean R. A. (1995). The cAMP-dependent protein kinase catalytic subunit is required for appressorium formation and pathogenesis by the rice blast pathogen *Magnaporthe grisea*. Plant Cell 7, 1869–1878. doi: 10.1105/tpc.7.11.1869, PMID: 8535140 PMC161045

[ref28] Nishimura M. Park G. Xu J. R. (2003). The G-beta subunit *MGB1* is involved in regulating multiple steps of infection-related morphogenesis in *Magnaporthe grisea*. Mol. Microbiol. 50, 231–243. doi: 10.1046/j.1365-2958.2003.03676.x, PMID: 14507377

[ref29] Park G. Xue C. Zhao X. Kim Y. Orbach M. Xu J. R. (2006). Multiple upstream signals converge on the adaptor protein Mst50 in *Magnaporthe grisea*. Plant Cell 18, 2822–2835. doi: 10.1105/tpc.105.038422, PMID: 17056708 PMC1626611

[ref30] Park G. Xue C. Zheng L. Lam S. Xu J. R. (2002). *MST12* regulates infectious growth but not appressorium formation in the rice blast fungus *Magnaporthe grisea*. Mol. Plant Microbe Interact. 15, 183–192. doi: 10.1094/MPMI.2002.15.3.183, PMID: 11952120

[ref31] Philips J. Herskowitz I. (1998). Identification of Kel1p, a kelch domain-containing protein involved in cell fusion and morphology in *Saccharomyces cerevisiae*. J. Cell Biol. 143, 375–389. doi: 10.1083/jcb.143.2.375, PMID: 9786949 PMC2132843

[ref32] Prag S. Adams J. C. (2003). Molecular phylogeny of the kelch-repeat superfamily reveals an expansion of BTB/kelch proteins in animals. BMC Bioinform. 4:42. doi: 10.1186/1471-2105-4-42, PMID: 13678422 PMC222960

[ref33] Qi L. L. Kim Y. Jiang C. Li Y. Peng Y. L. Xu J. R. (2015). Activation of Mst11 and feedback inhibition of germ tube growth in *Magnaporthe oryzae*. Mol. Plant Microbe Interact. 28, 881–891. doi: 10.1094/MPMI-12-14-0391-R, PMID: 26057388

[ref34] Qu Y. Wang J. Zhu X. Dong B. Liu X. Lu J.. (2020). The P5-type ATPase Spf1 is required for development and virulence of the rice blast fungus *Pyricularia oryzae*. Curr. Genet. 66, 385–395. doi: 10.1007/s00294-019-01030-5, PMID: 31471638

[ref35] Ramanujam R. Naqvi N. I. (2010). PdeH, a high-affinity camp phosphodiesterase, is a key regulator of asexual and pathogenic differentiation in *Magnaporthe oryzae*. PLoS Pathog. 6:e1000897. doi: 10.1371/journal.ppat.1000897, PMID: 20463817 PMC2865543

[ref36] Ryder L. S. Dagdas Y. F. Kershaw M. J. Venkataraman C. Madzvamuse A. Yan X.. (2019). A sensor kinase controls turgor-driven plant infection by the rice blast fungus. Nature 574, 423–427. doi: 10.1038/s41586-019-1637-x, PMID: 31597961

[ref37] Ryder L. S. Talbot N. J. (2015). Regulation of appressorium development in pathogenic fungi. Curr. Opin. Plant Biol. 26, 8–13. doi: 10.1016/j.pbi.2015.05.013, PMID: 26043436 PMC4781897

[ref38] Sabnam N. Barman S. R. (2017). *WISH*, a novel *CFEM GPCR* is indispensable for surface sensing, asexual and pathogenic differentiation in rice blast fungus. Fungal Genet. Biol. 105, 37–51. doi: 10.1016/j.fgb.2017.05.006, PMID: 28576657

[ref39] Sakulkoo W. Oses-Ruiz M. Garcia E. O. Soanes D. M. Littlejohn G. R. Hacker C.. (2018). A single fungal MAP kinase controls plant cell-to-cell invasion by the rice blast fungus. Science 359, 1399–1403. doi: 10.1126/science.aaq0892, PMID: 29567712

[ref40] Selvaraj P. Shen Q. Yang F. Naqvi N. I. (2017). Cpk2, a catalytic subunit of cyclic AMP-PKA, regulates growth and pathogenesis in rice blast. Front. Microbiol. 8:2289. doi: 10.3389/fmicb.2017.02289, PMID: 29209297 PMC5702331

[ref41] Shi Y. Wang H. Yan Y. Cao H. Liu X. Lin F.. (2018). Glycerol-3-phosphate shuttle is involved in development and virulence in the rice blast fungus *Pyricularia oryzae*. Front. Plant Sci. 9:687. doi: 10.3389/fpls.2018.00687, PMID: 29875789 PMC5974175

[ref42] Tafforeau L. Le Blastier S. Bamps S. Dewez M. Vandenhaute J. Hermand D. (2006). Repression of ergosterol level during oxidative stress by fission yeast F-box protein Pof14 independently of SCF. EMBO J. 25, 4547–4556. doi: 10.1038/sj.emboj.7601329, PMID: 17016471 PMC1589992

[ref43] Talbot N. J. (2003). On the trail of a cereal killer: exploring the biology of *Magnaporthe grisea*. Annu. Rev. Microbiol. 57, 177–202. doi: 10.1146/annurev.micro.57.030502.090957, PMID: 14527276

[ref44] Talbot N. J. Kershaw M. J. Wakley G. E. Devries O. M. H. Wessels J. G. H. Hamer J. E. (1996). *MPG1* encodes a fungal hydrophobin involved in surface interactions during infection-related development of *Magnaporthe grisea*. Plant Cell 8, 985–999. doi: 10.2307/3870210, PMID: 12239409 PMC161153

[ref45] Tang Q. Y. Zhang C. X. (2013). Data processing system (DPS) software with experimental design, statistical analysis and data mining developed for use in entomological research. Insect Sci. 20, 254–260. doi: 10.1111/j.1744-7917.2012.01519.x, PMID: 23955865

[ref46] Thines E. Weber R. W. S. Talbot N. J. (2000). MAP kinase and protein kinase A-dependent mobilization of triacylglycerol and glycogen during appressorium turgor generation by *Magnaporthe grisea*. Plant Cell 12, 1703–1718. doi: 10.1105/tpc.12.9.1703, PMID: 11006342 PMC149080

[ref47] Toda T. Uno I. Ishikawa T. Powers S. Kataoka T. Broek D.. (1985). In yeast, *RAS* proteins are controlling elements of adenylate-cyclase. Cell 40, 27–36. doi: 10.1016/0092-8674(85)90305-8, PMID: 2981630

[ref48] Tsuji G. Kenmochi Y. Takano Y. Sweigard J. Farrall L. Furusawa I.. (2000). Novel fungal transcriptional activators, Cmr1p of *Colletotrichum lagenarium* and Pig1p of *Magnaporthe grisea*, contain Cys2His2 zinc finger and Zn(II)2Cys6 binuclear cluster DNA-binding motifs and regulate transcription of melanin biosynthesis genes in a developmentally specific manner. Mol. Microbiol. 38, 940–954. doi: 10.1046/j.1365-2958.2000.02181.x, PMID: 11123670

[ref49] Vo T. V. Das J. Meyer M. J. Cordero N. A. Akturk N. Wei X. M.. (2016). A proteome-wide fission yeast interactome reveals network evolution principles from yeasts to human. Cell 164, 310–323. doi: 10.1016/j.cell.2015.11.037, PMID: 26771498 PMC4715267

[ref50] Wang J. Y. Guo X. Y. Li L. Qiu H. P. Zhang Z. Wang Y. L.. (2018). Application of the fluorescent dye BODIPY in the study of lipid dynamics of the rice blast fungus *Magnaporthe oryzae*. Molecules 23:1594. doi: 10.3390/molecules23071594, PMID: 29966327 PMC6099410

[ref51] Wei Y. Y. Liang S. Zhang Y. R. Lu J. P. Lin F. C. Liu X. H. (2020). MoSec61β, the beta subunit of Sec61, is involved in fungal development and pathogenicity, plant immunity, and ER-phagy in *Magnaporthe oryzae*. Virulence 11, 1685–1700. doi: 10.1080/21505594.2020.1848983, PMID: 33200669 PMC7714445

[ref52] Wilson R. A. Talbot N. J. (2009). Under pressure: investigating the biology of plant infection by *Magnaporthe oryzae*. Nat. Rev. Microbiol. 7, 185–195. doi: 10.1038/nrmicro2032, PMID: 19219052

[ref53] Xu J. R. Hamer J. E. (1996). MAP kinase and cAMP signaling regulate infection structure formation and pathogenic growth in the rice blast fungus *Magnaporthe grisea*. Genes Dev. 10, 2696–2706. doi: 10.1101/gad.10.21.2696, PMID: 8946911

[ref54] Xu J. R. Urban M. Sweigard J. A. Hamer J. E. (1997). The *CPKA* gene of *Magnaporthe grisea* is essential for appressorial penetration. Mol. Plant Microbe Interact. 10, 187–194. doi: 10.1094/MPMI.1997.10.2.187

[ref55] Yan Y. X. Wang H. Zhu S. Y. Wang J. Liu X. H. Lin F. C.. (2019). The methylcitrate cycle is required for development and virulence in the rice blast fungus *Pyricularia oryzae*. Mol. Plant Microbe Interact. 32, 1148–1161. doi: 10.1094/MPMI-10-18-0292-R, PMID: 30933666

[ref56] Zhang X. Bian Z. Xu J. R. (2018). Assays for MAP kinase activation in *Magnaporthe oryzae* and other plant pathogenic fungi. Methods Mol. Biol. 1848, 93–101. doi: 10.1007/978-1-4939-8724-5_8, PMID: 30182231

[ref57] Zhao X. H. Kim Y. Park G. Xu J. R. (2005). A mitogen-activated protein kinase cascade regulating infection-related morphogenesis in *Magnaporthe grisea*. Plant Cell 17, 1317–1329. doi: 10.1105/tpc.104.029116, PMID: 15749760 PMC1088005

[ref58] Zhao X. H. Xu J. R. (2007). A highly conserved MAPK-docking site in Mst7 is essential for Pmk1 activation in *Magnaporthe grisea*. Mol. Microbiol. 63, 881–894. doi: 10.1111/j.1365-2958.2006.05548.x, PMID: 17214742

[ref59] Zhou X. Y. Zhao X. H. Xue C. Y. Dai Y. F. Xu J. R. (2014). Bypassing both surface attachment and surface recognition requirements for appressorium formation by overactive Ras signaling in *Magnaporthe oryzae*. Mol. Plant Microbe Interact. 27, 996–1004. doi: 10.1094/MPMI-02-14-0052-R, PMID: 24835254

[ref60] Zhu S. Yan Y. Qu Y. Wang J. Feng X. Liu X.. (2020). Role refinement of melanin synthesis genes by gene knockout reveals their functional diversity in *Pyricularia oryzae* strains. Microbiol. Res. 242:126620. doi: 10.1016/j.micres.2020.126620, PMID: 33189072

