# Supplementary material for: PoRal2 Is Involved in Appressorium Formation and Virulence via Pmk1 MAPK Pathways in the Rice Blast Fungus Pyricularia oryzae
Source: Front Plant Sci. 2021 Sep 13;12:702368. doi: 10.3389/fpls.2021.702368 (PMC8473790; doi:10.3389/fpls.2021.702368)
Supplement: Supplementary file 1 [file Data_Sheet_1.PDF]

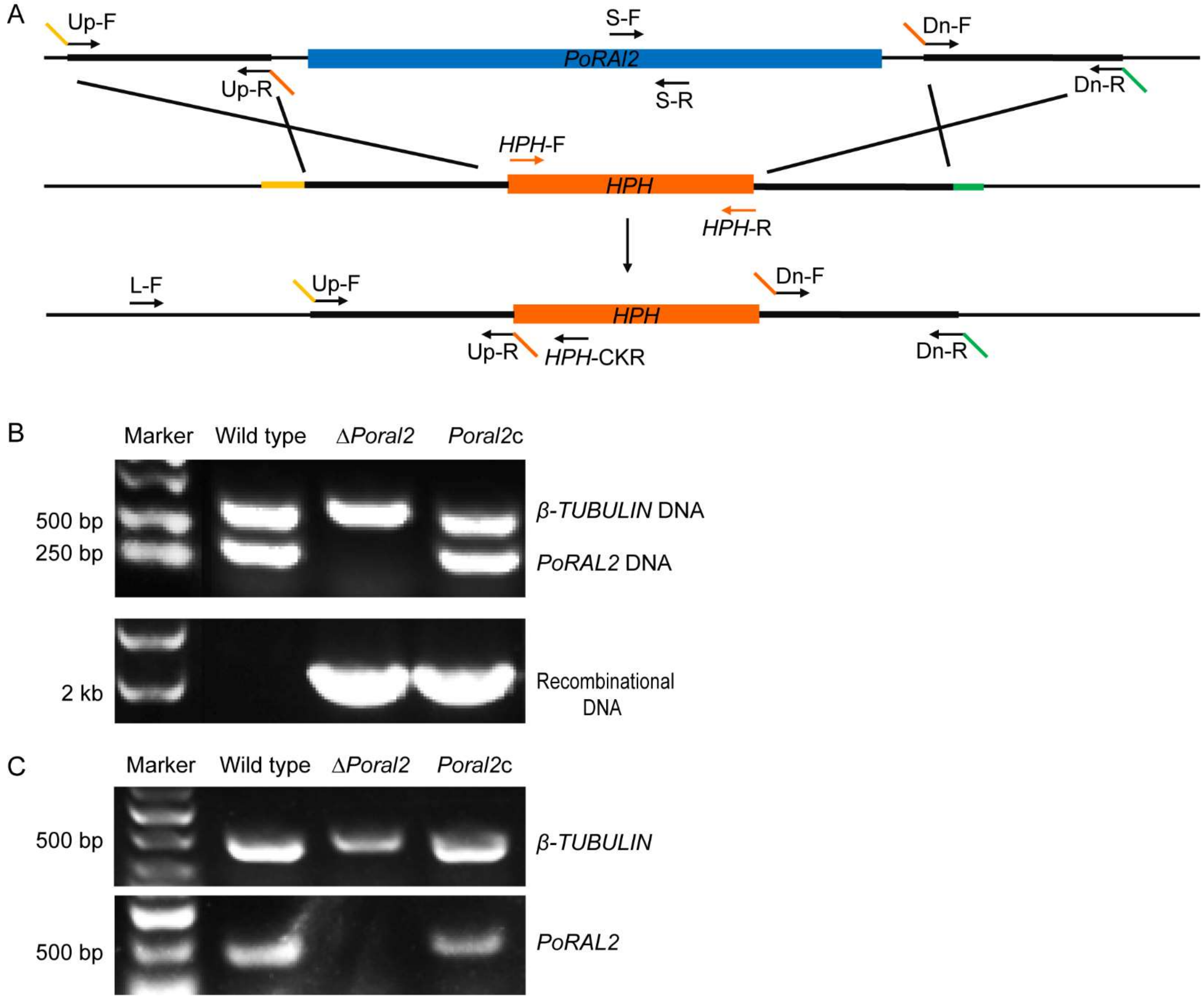

Supplementary FIGURE S1 Knockout and complementation of *PoRAL2* in *P. oryzae* strain 70-15. (**A**) Knockout strategy. Up-F/Up-R and Dn-F/Dn-R are the two primer sets used to clone two flanking fragments of *PoRAL2*. The L-F/*HPH*-CKR primer set was used to clone the recombinant DNA fragment in null mutants. The S-F/S-R primer set was used to clone a partial fragment of *PoRAL2* in the wild type and transformants. (**B**) Knockout events were confirmed at the DNA level. The Δ*Poral2* strain only showed a PCR band of 500 bp (representing *β-TUBULIN*, used as a positive control), while the wild type also had a band of 307 bp for *PoRAL2* (upper panel). Δ*Poral2* had a ~2 kb-long recombinant DNA band, while the wild type did not (lower panel). (**C**) Complementation of Δ*Poral2* by native *PoRAL2*. The mRNA expression level of *PoRAL2* in the complemented strain *Poral2*c was confirmed by RT-PCR. *β-TUBULIN* was used as a control.
