# Supplementary material for: PoRal2 Is Involved in Appressorium Formation and Virulence via Pmk1 MAPK Pathways in the Rice Blast Fungus Pyricularia oryzae
Source: Front Plant Sci. 2021 Sep 13;12:702368. doi: 10.3389/fpls.2021.702368 (PMC8473790; doi:10.3389/fpls.2021.702368)
Supplement: Supplementary file 2 [file Data_Sheet_2.PDF]

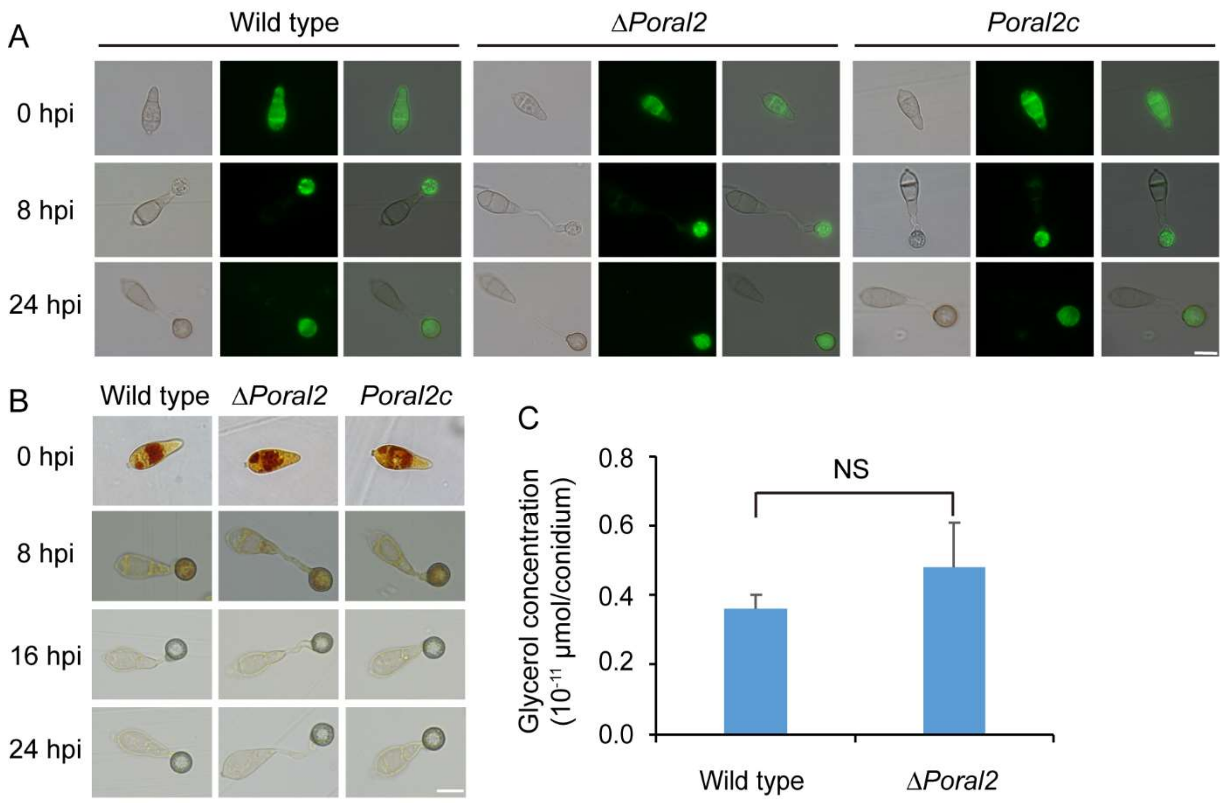

Supplementary FIGURE S2 Lipid droplet and glycogen translocation and degradation in *P. oryzae* strains. (**A-B**) Conidia and appressoria at 0, 8, 24 hpi of the wild type, Δ*Poral2* and *Poral2c* strains were stained with Boron dipyrromethene (BODIPY) to detect lipid droplets, or with Iodine solution containing 60 mg/ml KI and 10 mg/ml I_2 to detect glycogen and observed under fluorescence microscope. Bar = 10 μm. (**C**) The glycerol concentration of appressoria at 24 hpi was measured using the Glycerin content GPO-POD enzymatic assay kit (Applygene, China) in triplicate. Error bars represent standard deviations. Significant differences compared with the wild type were estimated by Tukey's HSD test: **P* < 0.05 and ***P* < 0.01.
