# Supplementary material for: PoRal2 Is Involved in Appressorium Formation and Virulence via Pmk1 MAPK Pathways in the Rice Blast Fungus Pyricularia oryzae
Source: Front Plant Sci. 2021 Sep 13;12:702368. doi: 10.3389/fpls.2021.702368 (PMC8473790; doi:10.3389/fpls.2021.702368)
Supplement: Supplementary file 3 [file Data_Sheet_3.PDF]

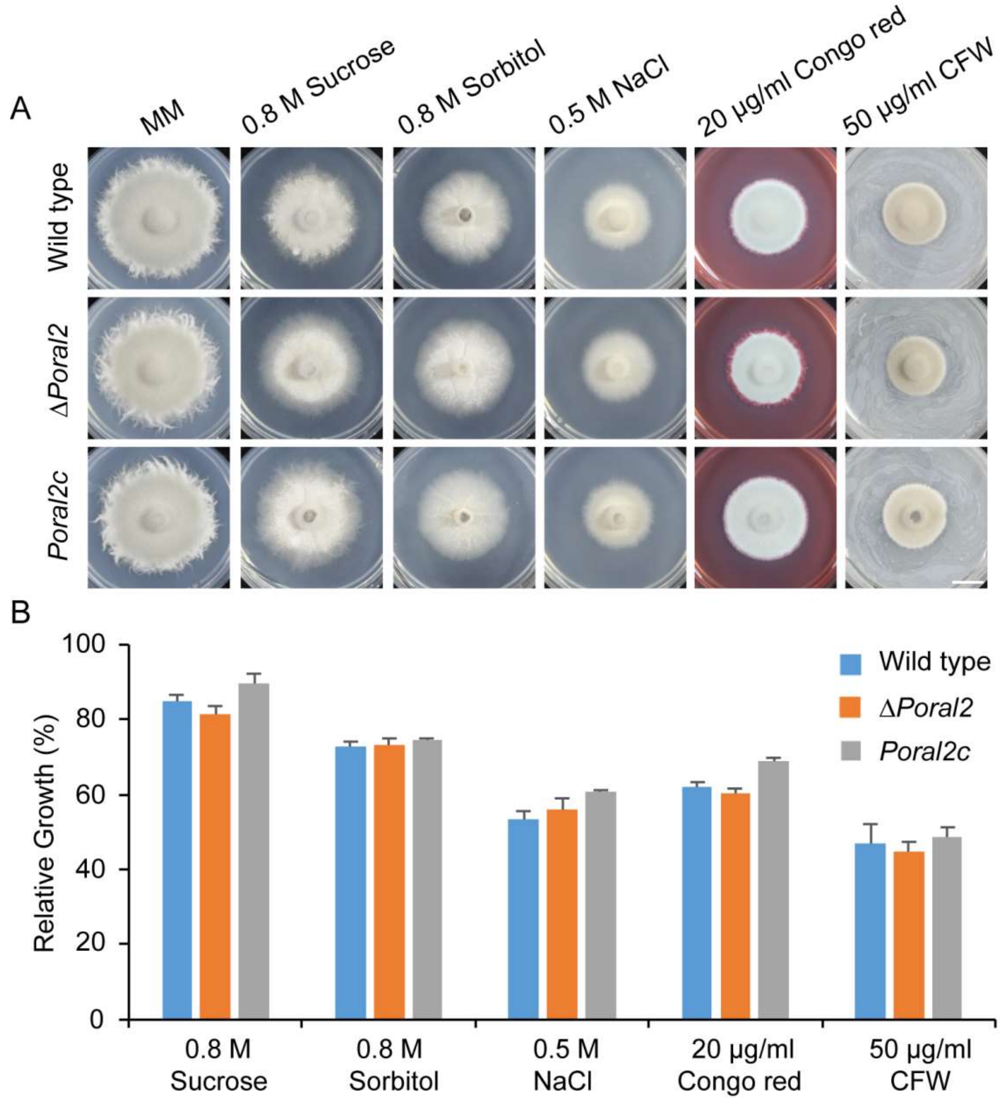

Supplementary FIGURE S3 Responses of *P. oryzae* strains to osmotic and cell wall stresses. Mycelial colonies (**A**) and relative growth (%) (**B**) of the wild type, Δ*Poral2*, and *Poral2c* strains cultured on MM media containing 0.8 M Sucrose, 0.8 M Sorbitol, 0.5 M NaCl, 20 μg/ml Congo Red and 50 μg/ml CFW under darkness for 10 days at 25° C. Error bars represent standard deviations. Significant differences compared with the wild type were estimated by Tukey's HSD test: *$P < 0.05$ and **$P < 0.01$.
