# Supplementary figures and images for: PoRal2 Is Involved in Appressorium Formation and Virulence via Pmk1 MAPK Pathways in the Rice Blast Fungus Pyricularia oryzae
Source: Front Plant Sci. 2021 Sep 13;12:702368. doi: 10.3389/fpls.2021.702368 (PMC8473790; doi:10.3389/fpls.2021.702368)

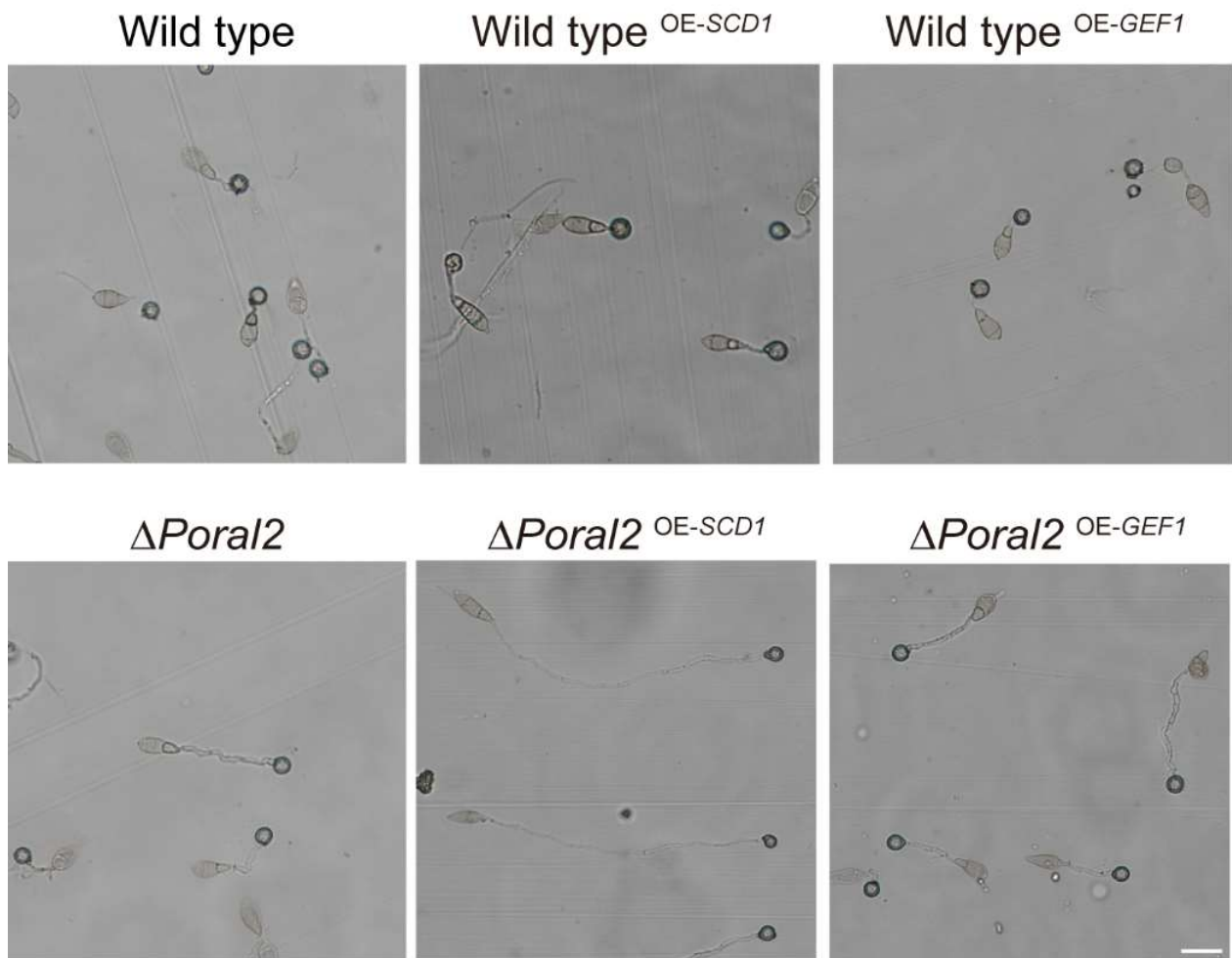

**Supplementary FIGURE S4** Appressorium formation of  $\Delta Poral2$  overexpressing *SCD1* or *GEF1*.

Supplement: Supplementary file 4 [file Data_Sheet_4.PDF]
