# Supplementary material for: PoRal2 Is Involved in Appressorium Formation and Virulence via Pmk1 MAPK Pathways in the Rice Blast Fungus Pyricularia oryzae
Source: Front Plant Sci. 2021 Sep 13;12:702368. doi: 10.3389/fpls.2021.702368 (PMC8473790; doi:10.3389/fpls.2021.702368)
Supplement: Supplementary file 5 [file Data_Sheet_5.PDF]

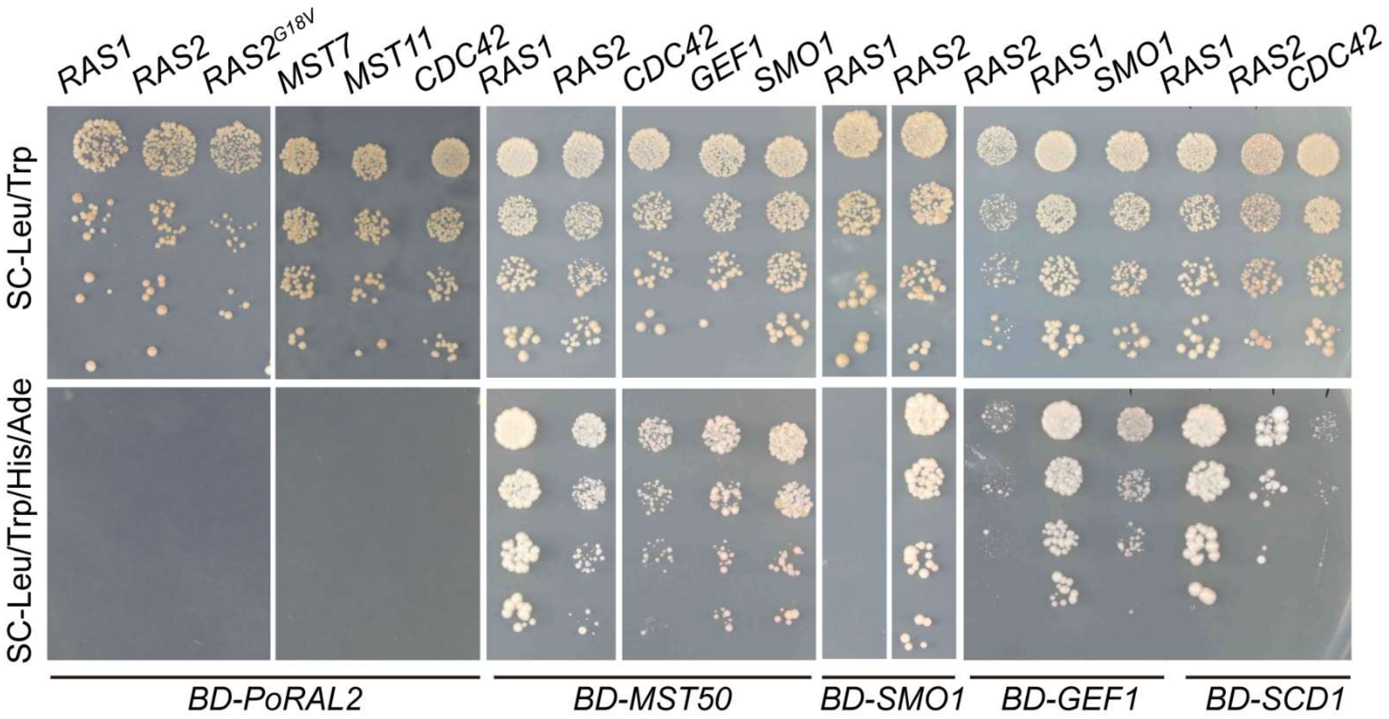

Supplementary FIGURE S5 Interactions among PoRal2, Mst50, Smo1, Scd1, Gef1, Ras1, Ras2 and Cdc42 by yeast two-hybrid assays. Yeast co-transformed with PoRal2 and Ras1, Ras2, Ras2^{G18V}, Mst7, Mst11 or Cdc42 failed to grow on SD-Leu-Trp-Ade-His medium. Yeast co-transformed with Mst50 and Ras1, Ras2, Cdc42, Gef1, SMO1, with Smo1 and Ras2, with Gef1 and Ras2, Ras2, Smo1, and with Ras1, Ras2, Cdc42 grew on SD-Leu-Trp-Ade-His medium.
