# Supplementary material for: PoRal2 Is Involved in Appressorium Formation and Virulence via Pmk1 MAPK Pathways in the Rice Blast Fungus Pyricularia oryzae
Source: Front Plant Sci. 2021 Sep 13;12:702368. doi: 10.3389/fpls.2021.702368 (PMC8473790; doi:10.3389/fpls.2021.702368)
Supplement: Supplementary file 6 [file Data_Sheet_6.PDF]

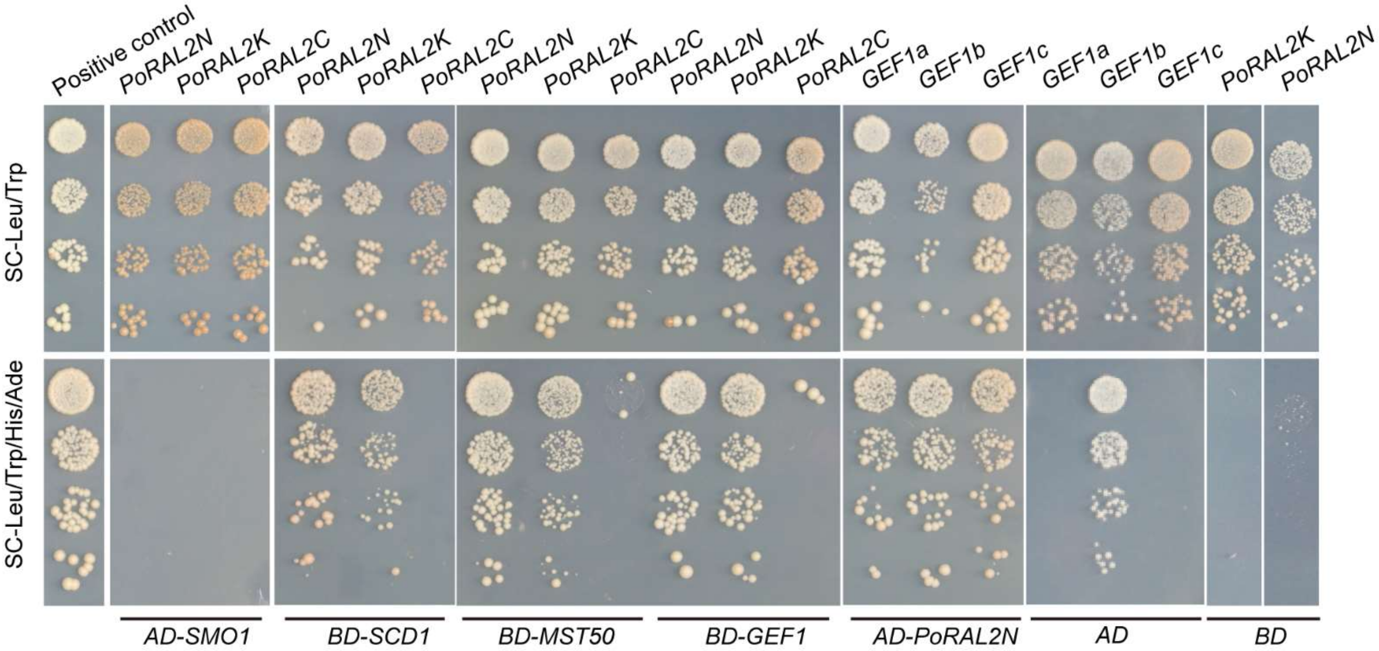

Supplementary FIGURE S6 Interactions of Smo1, Scd1, Mst50 and Gef1 with PoRal2N, PoRal2K and PoRal2C in Y2HGold. Smo1 co-transformed with PoRal2N, PoRal2K or PoRal2C could not grow on SD-Leu-Trp-Ade-His medium. Scd1, Mst50, and Gef1 co-transformed with PoRal2N, PoRal2K but not with PoRal2C could grow on SD-Leu-Trp-Ade-His medium. Gef1a and Gef1c co-transformed with PoRal2N grew on SD-Leu-Trp-Ade-His medium, while the Gef1b showed strong self-activation.
