# Supplementary material for: PoRal2 Is Involved in Appressorium Formation and Virulence via Pmk1 MAPK Pathways in the Rice Blast Fungus Pyricularia oryzae
Source: Front Plant Sci. 2021 Sep 13;12:702368. doi: 10.3389/fpls.2021.702368 (PMC8473790; doi:10.3389/fpls.2021.702368)
Supplement: Supplementary file 7 [file Data_Sheet_7.pdf]

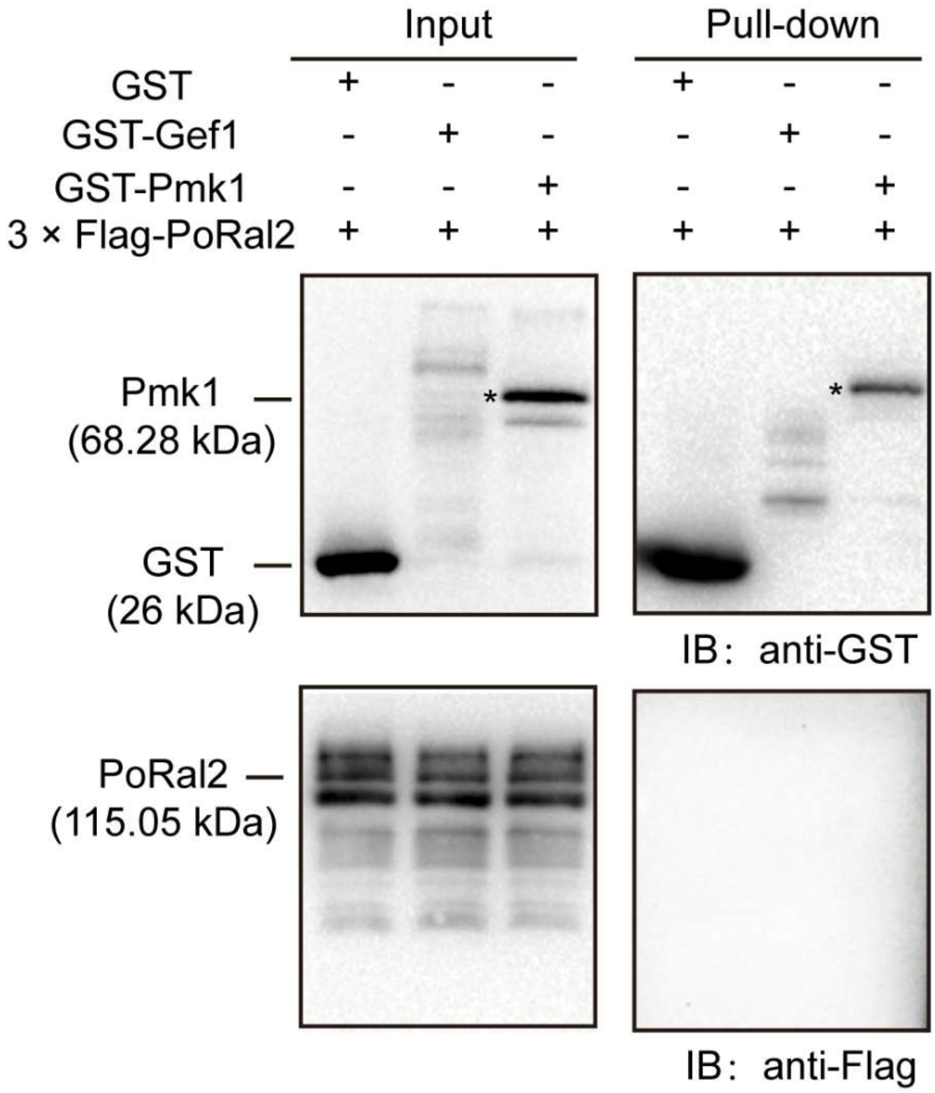

Supplementary FIGURE S7 Pull-down results of Flag-PoRal2 with GST, GST-Gef1 and GST-Pmk1 in *E. coli* BL21. GST-Gef1 failed to be identified, and Pmk1 failed to pull down 3 x Flag-PoRal2 by pull-down assays. Asterisks represented the band of GST-Pmk1.
