# Supplementary material for: PoRal2 Is Involved in Appressorium Formation and Virulence via Pmk1 MAPK Pathways in the Rice Blast Fungus Pyricularia oryzae
Source: Front Plant Sci. 2021 Sep 13;12:702368. doi: 10.3389/fpls.2021.702368 (PMC8473790; doi:10.3389/fpls.2021.702368)
Supplement: Supplementary file 8 [file Data_Sheet_8.docx]

**Table S1** Primers used in this study

| 1. Primers used to build gene deletion cassettes | | | | | |
| --- | --- | --- | --- | --- | --- |
| Up-F | GAGGCTAACTGACACTCTAGAGACTGATTCCTGGAGACAAACC | | | | |
| Up-R | CATTCATTGTTGACCTCCACTAGTAGGTGGATGTCGGGTAAAG | | | | |
| Dn-F | GGGCAAAGGAATAGAGTAGATGTCATCGCCTCTGAACTTGTG | | | | |
| Dn-R | TGTAAAACGACGGCCAGTGCCACTGACGGTTCCCTTGTCTATG | | | | |
| *HPH*-F | TAGTGGAGGTCAACAATGAATG | | | | |
| *HPH*-R | CATCTACTCTATTCCTTTGCCC | | | | |
| 2 Primers used to identify ∆*Poral2* and *Poral2c* | | | | | |
| S-F | ACAAGAATACGCGCTATGGG | | | | |
| S-R | GCTCAAGCCAGGACTTGTATAG | | | | |
| L-F | ATCTCCGTGGCACTTCCGTAA | | | | |
| *HPH*-CKR | GGGCGAACTTAAGAAGGTATGA | | | | |
| Tbl-gF | TTCCGCGCTGTCACCGTTCC | | | | |
| Tbl-gR | GGGCCTCCTCCTCGTACTCCTCTT | | | | |
| c*Poral2*-F | GCTCTTCGATGCTCATCTACTC | | | | |
| c*Poral2*-R | GTAGTCGTCCACCAAACTTCTT | | | | |
| qtub-F | ACAACTTCGTCTTCGGTCAG | | | | |
| qtub-R | GTGATCTGGAAACCCTGGAG | | | | |
| q*HPH*-F | ATGTCCTGCGGGTAAATAGC | | | | |
| q*HPH*-R | GATGCAATAGGTCAGGCTCTC | | | | |
| 3 Primers used in the complementation and fluorescent experiments | | | | | |
| *PoRAL2*c-F | CACAATCACTAGTGAATTCTCTAATGGCTTTGCTGCGA | | | | |
| *PoRAL2*c-R | GTCGCTTACTGCAGGTCGACCATCACAGAACTCCAGGACATG | | | | |
| GFP-*RAS2*-F | CATGGACGAGCTGTACAAGATGGCTCAGTCAAAGGTTCG | | | | |
| GFP-*RAS2*-R | CTGCAGGTCGACTCTAGATTACATCAAGACACACTTGG | | | | |
| GFP-*RAS2*^G18V^up-R | AAGCATGATTTACCGACACCGACGCCGCCGACAACCACCAGTT | | | | |
| GFP-*RAS2*^G18V^dn-F | GGTGTCGGTAAATCATGCTT | | | | |
| *BAS4*-mCherry-F | CACCGAGATTTAGGAATTCGGTAGCTTCTACGGATGCGTCCGATG | | | | |
| *BAS4*-mCherry-R | GCTCACCATCCCGGGGATGGATCCAGCAGGGGGGATAGACGAGCCAGTAG | | | | |
| *PWL2*- mCherry-NLS-F | GCTCTCACGATTCACAATCACTAGTGAATTCGTCGCGTCAGTGAACAAACCTGT | | | | |
| *PWL2*- mCherry-NLS-R | GCTCACCATCCCGGGGATGGATCCCATAATATTGCAGCCCTCTTC | | | | |
| *PoRAL2N-GFP-F* | ATCACAATGGCCGGATCCATGAGCAACGCAGGCTCTCG | | | | |
| *PoRAL2N-GFP-R* | AGCTCCTCGCCCTTGCTCACGCCGCCGCCGCCGCCCGGCGTATATGCTCCAGACA | | | | |
| *MST50-3×FLAG-F* | CAATCACAATGGCCGGATCCATGAGCTTCAACACGGGGAC | | | | |
| *MST50-3×FLAG-R* | TGGTCCTTGTAGTCCCCGGGTATTATTCCTCCTGGGGGAT | | | | |
| *3×FLAG* -*SCD1*-F | ATGATGACGACAAGTCTAGAATGGCATATGCGCCCCTCTTG | | | | |
| *3×FLAG* -*SCD1*-R | CGCTTACTGCAGGTCGACCTACTCGCCACCAATGCCAAC | | | | |
| *SMO1*-*3×FLAG-*F | ATGATGACGACAAGTCTAGAATGTCCGTCATGCTGCAAAC | | | | |
| *SMO1*-*3×FLAG-*R | TCGCTTACTGCAGGTCGACCTACCAGCCCTTCTTCCGCG | | | | |
| *3×FLAG* -*GEF1*-F | ATGATGACGACAAGTCTAGAATGGACGGGGGCAACGAGGAG | | | | |
| *3×FLAG* -*GEF1*-R | CGCTTACTGCAGGTCGACCTATGGGTCTGCAAGCTTCG | | | | |
| 4 Primers used in qPCR | | | | | |
| *MPG1*-qF | MGG_10315  MGG_09499 | | | AGTGCAAGAACATCCCGATC | |
| *MPG1*-qR  *RAS1*-qF  *RAS1*-qR |  |  |  | GTGCACTGGATGTTGACCA  CACAAGCTGGTAGTCCTCG  AAGCCTGGTTGTCTATGACG | |
| *RAS*2-qF | MGG_06154 | | | GTAACAAGTGCGATCTGGAGG | |
| *RAS*2-qR |  |  |  | CGTTGATTCTTGACTTTGCCG | |
| *PTH11*-qF | MGG_05871 | | | GTTCGGAATGCTTGGAATGC | |
| *PTH11*-qR |  |  |  | AAGACCTGGACCACAATGTAC | |
| *PMK1*-qF | MGG_09565 | | | ATTTCCATCCTCGACATCCAG | |
| *PMK1*-qR |  |  |  | GTCTGGTAGATGAAGTACTGGC | |
| *OSM1*-qF | MGG_01822 | | | CACCTGAAGCACGAAAATGTC | |
| *OSM1*-qR |  |  |  | AAGGAAGTACTGGATGAACTGC | |
| *WISH1*-qF | MGG_09022 | | | AGTTCTGGCCCTGTTTACG | |
| *WISH1*-qR |  |  |  | CATTCGCTCATACGATCCAGG | |
| *PDEH*-qF | MGG_05664 | | | AATTCTGACGGAACTGAGGG | |
| *PDEH*-qR |  |  |  | CAAGTCGAAGAGCTGGGTC | |
| *BUF1*-qF | MGG_02252 | | | TGTCTTTACCATCAACACCCG | |
| *BUF1*-qR |  |  |  | CTTTGATCCTGAGTAGACGGC | |
| *PKC1*-qF | MGG_08689 | | | CCCCGAGTTTATGGCACC | |
| *PKC1*-qR |  |  |  | CTCATCTTCGTCTTCACCTCTG | |
| *MAC1*-qF | MGG_09898 | | | AGACCGCAACTATTCCAACG | |
| *MAC1*-qR |  |  |  | CAGGAGCTTCGCCATATTTTG | |
| *RGS7*-qF | MGG_11693 | | | GAGAGAAGGATTGTGGTCGTC | |
| *RGS7*-qR |  |  |  | GTGGTATTTGCGGGAGAGG | |
| *RGS1*-qF | MGG_14517 | | | TGGTTGAGACATTGCAGGAG | |
| *RGS1*-qR |  |  |  | AAGTCGTAGTTCTTGAGTAGATGC | |
| *ALB1-*qF | MGG_07219 | | | ACTAAACGAGCGGTATCATGC | |
| *ALB1-*qR |  |  |  | GGTAGGTTTTGTCATGCTGTG | |
| *PIG1-*qF | MGG_07215 | | | TGTGGACAGAAGTTTACTCGC | |
| *PIG1-*qR |  |  |  | CTCGTGGTATGCTTGGTAGTG | |
| *RSY1*-qF  *RSY1-*qR | MGG_05059 | | | GTTCAAAAGAGCGATGAGATAACC  GGAAGGAGCGGTAGTCAATG | |
| 5 Primers used in yeast two-hybrid | | | | | |
| *PoRAl2-*ADF | | GGAGGCCAGTGAATTCATGAGCAACGCAGGCTCTC | | | |
| *PoRAl2-*ADR | | CGAGCTCGATGGATCCGTTACCACCGGCGATACC | | | |
| *PoRAl2-*BDF | | GCCATGGAGGCCGAATTCATGAGCAACGCAGGCTCTCG | | | |
| *PoRAl2-*BDR | | CTGCAGGTCGACGGATCCGTATCGCCGGTGGTAACTAG | | | |
| *RAS1*-ADF | | GGAGGCCAGTGAATTCATGACTGGAAGGTTGCAGCT | | | |
| *RAS1*-ADR | | CGAGCTCGATGGATCCTCACAATATAACACACTTGA | | | |
| *RAS2*-ADF | | GGAGGCCAGTGAATTCATGGCTCAGTCAAAGTTCCTACG | | | |
| *RAS2*-ADR | | CGAGCTCGATGGATCCCATCAAGACACACTTGGAGCAG | | | |
| *RAS2*^G18V^*-*ADF | | ATGGCTCAGTCAAAGTTCCTACGCGAGTACAAACTGGTTGTCGTAGGAGGTGTAGGTGTCGGTAAATCATGCTT | | | |
| *CDC42*-ADF | | GGAGGCCAGTGAATTCATGGTGGTTGCAACGATT | | | |
| *CDC42*-ADR | | CGAGCTCGATGGATCCAAGGATCAGGCACTTTTTG | | | |
| *GEF1*-ADF | | GGAGGCCAGTGAATTCATGGACGGGGGCAACGAG | | | |
| *GEF1*-ADR | | CGAGCTCGATGGATCCTGGGTCTGCAAGCTTCGCAAAAT | | | |
| *SCD1*-BDF | | GCCATGGAGGCCGAATTCATGGCATATGCGCCCCTCTTG | | | |
| *SCD1*-BDR | | CTGCAGGTCGACGGATCCCTACTCGCCACCAATGCCAAC | | | |
| *GEF1*-BDF | | GCCATGGAGGCCGAATTCATGGACGGGGGCAACGAGGAG | | | |
| *GEF1*-BDR | | CTGCAGGTCGACGGATCCCTATGGGTCTGCAAGCTTCG | | | |
| *MST50*-BDF | | GCCATGGAGGCCGAATTCATGAGCTTCAACACGGGGAC | | | |
| *MST50*-BDR | | CTGCAGGTCGACGGATCCTCATATTATTCCTCCTGGGG | | | |
| *SMO1*-ADF | | GGAGGCCAGTGAATTCATGTCCGTCATGCTGCAAACTC | | | |
| *SMO1*-ADR | | CGAGCTCGATGGATCCCCAGCCCTTCTTCCGCGCGAAC | | | |
| *SMO1*-BDF | | GCCATGGAGGCCGAATTCATGTCCGTCATGCTGCAAACTC | | | |
| *SMO1*-BDR | | CTGCAGGTCGACGGATCC CTACCAGCCCTTCTTCCGCG | | | |
| *MST11*-BDF | | GCCATGGAGGCCGAATTC ATGGCCATGTTGGCTTCAAA | | | |
| *MST11*-BDR  *MST7-*ADF  *MST7*-ADR  *PMK1*-ADF  *PMK1*-ADR | | CTGCAGGTCGACGGATCCTTACGTGATAGGAGTCAGGA  GGAGGCCAGTGAATTCATGGCCGACCCGTTTGCG  CGAGCTCGATGGATCCGTCCCGGATGTAAAGCCGATC  GGAGGCCAGTGAATTCATGTCTCGCGCCAATCCACC  CGAGCTCGATGGATCCTTACCGCATAATTTCCTGGT | | | |
| *PoRAl2N-*BDR | | CTGCAGGTCGACGGATCCCGGCGTATATGCTCCAGACA | | | |
| *Po RAl2K-*BDF | | GCCATGGAGGCCGAATTCGGAGCAACCACAACCGTGC | | | |
| *Po RAl2K-*BDR | | CTGCAGGTCGACGGATCCGAGTGGCTGCGTCGAGGTCC | | | |
| *Po RAl2C-*BDF | | GCCATGGAGGCCGAATTCCCTGGACTTCGTTTCCCCAA | | | |
| *Po RAl2N-*ADR | | AGCTCGAGCTCGATGGATCCCGGCGTATATGCTCCAGACA | | | |
| *Po RAl2K-*ADF | | CCATGGAGGCCAGTGAATTCGGAGCAACCACAACCGTGC | | | |
| *Po RAl2K-*ADR | | AGCTCGAGCTCGATGGATCCGAGTGGCTGCGTCGAGGTCC | | | |
| *Po RAl2C-*ADF | | CCATGGAGGCCAGTGAATTCCCTGGACTTCGTTTCCCCAA | | | |
| *GEF1a-*BDF | | GCCATGGAGGCCGAATTCATGGACGGGGGCAACGAGGA | | | |
| *GEF1a-*BDR | | CTGCAGGTCGACGGATCCTTGAACTGCGCCCTCTGCCC | | | |
| *GEF1b-*BDF | | GCCATGGAGGCCGAATTCCCCACGACTGATGCGACACA | | | |
| *GEF1b-*BDR | | CTGCAGGTCGACGGATCCGTTGATGTCAAGAATAGCAG | | | |
| *GEF1c-*BDF | | GCCATGGAGGCCGAATTCAAAACGAAGAAGAACTTCGA | | | |
| *GEF1c-*BDR | | CTGCAGGTCGACGGATCCCTATGGGTCTGCAAGCTTCG | | | |
| 6 Primers used in pull down assay | | | | | |
| pGEX4T-GST-*SCD1*-F | | | GGTTCCGCGTGGATCCCCGATGGCATATGCGCCCCTCTT | |  |
| pGEX4T -GST-*SCD1*-R | | | TCGAGTCGACCCGGGAATTCCTACTCGCCACCAATGCCAA | |  |
| pGEX4T -GST-*SMO1*-F | | | GGTTCCGCGTGGATCCCCGATGTCCGTCATGCTGCAAAC | |  |
| pGEX4T -GST-*SMO1*-R | | | TCGAGTCGACCCGGGAATTCCTACCAGCCCTTCTTCCGCG | |  |
| pGEX4T -GST-*MST50*-F | | | GGTTCCGCGTGGATCCCCGATGAGCTTCAACACGGGGACGGCGTACG | |  |
| pGEX4T -GST-*MST50*-R | | | TCGAGTCGACCCGGGAATTCTCATATTATTCCTCCTGGGGGATCATAT | |  |
| pGEX4T -GST-*GEF1*-F | | | TCGGATCTGGTTCCGCGTGGATCCCCGATGGACGGGGGCAACGAGGA | |  |
| pGEX4T -GST-*GEF1*-R | | | CGGCCGCTCGAGTCGACCCGGGAATTCCTATGGGTCTGCAAGCTTCG | |  |
| pGEX4T -GST-*PMK1*-F | | | GGTTCCGCGTGGATCCCCGATGTCTCGCGCCAATCCACC | |  |
| pGEX4T -GST-*PMK1*-R | | | TCGAGTCGACCCGGGAATTCTTACCGCATAATTTCCTGGT | |  |
| pET21-*PoRAL2*-F | | | TAAGGATGATGACGACAAG ATGAGCAACGCAGGCTCTCG | |  |
| pET21- *PoRAL2*-R | | | GTGGTGGTGGTGGTGGTGGTTACCACCGGCGATACCCA | |  |

**Table S2** Copy identification of the resistant gene (*HPH*) inserted in the mutant’s genome by qPCR

| Mutant No | Copies of *HPH* by qPCR | Insertion event |
| --- | --- | --- |
| ∆*Poral2* | 0.82^a^ | Single^b^ |

^a^Copies of the *HPH* gene in the mutant’s genome were quantified by qPCR and calculated after compared with the Southern blot-confirmed mutant.

^b^“Single” represents the targeted gene deletion event without ectopic insertion.

**Table S3** Relative expression level of *PoRAL2* in the wild type and *PoRAL2*-complemented strain of ∆*Poral2* (*Poral2****c*)** by qPCR

| Strain | Expression level (2^-∆CT^) |
| --- | --- |
| 70-15 | 0.05^a^ |
| *Poral2c* | 0.07 |

^a^PoRAL2 mRNA level is calculated relative to a reference gene *β*-*TUBULIN*.
